# Predicting energy consumption SAG mills through Bayesian generalized linear model and random forest

**DOI:** 10.1177/25726668251391540

**Published:** 2025-11-05

**Authors:** Zhanbolat Magzumov, Mustafa Kumral

**Affiliations:** Mining and Materials Engineering Department, 5620 McGill University, Montréal, QC, Canada

**Keywords:** Bayesian models, energy consumption, random forest, mineral processing, mine equipment, semi-autogenous grinding mill

## Abstract

The mining industry consumes about 1.7% of the energy generated worldwide, which is expected to increase in the coming decades. Milling is the most energy-intensive process of a typical mining operation. Many variables (e.g., rock characteristics, mineral matrix, and equipment properties) affect energy consumption. This paper proposes that Random Forests and the Generalised Linear Model (GLM) be used to predict the energy consumption of the SAG mill, which significantly contributes to the energy consumption of mining operations. To show the performance of the proposed approach, a case study was applied to a copper mine dataset from South America. The proposed approaches were applied to forecast the SAG mill energy consumption. The outcomes demonstrated that these methods could be used to predict energy consumption. Random Forest can have a high prediction accuracy of 95% but lacks explanatory ability, as shown in R2 at 50%. GLM provided additional insights by showing the feature importances and their relationships with SAG mill energy consumption, along with considering the potential uncertainties and generating posterior probability distributions for the model outcomes. Both models identified key variables as significant predictors identically, with the GLM offering a more comprehensive view of best-case and worst-case energy consumption scenarios.

## Introduction

Despite the finite availability of metals on Earth, our dependency on these materials and their wide-ranging applications continues to grow (Rötzer and Schmidt, 2018; Graedel et al., 2015). Mineral resources such as copper, nickel, cobalt, lithium, and rare earth elements are essential in various industries, including construction, electronics, and transportation, underscoring their ongoing relevance (Agosti et al., 2022).

The depletion of crucial metals, such as base metals, has been well-documented. Mudd (2007a, 2007b, 2010) has comprehensively analysed the dwindling reserves of these critical resources. Calvo et al. (2016) reported significant declines in ore grades from major copper producers such as Chile, Peru, and Australia. This decline in ore quality necessitates more energy-intensive and costlier extraction processes. In the decade between 2003 and 2013, ore grades dropped by a quarter, with most high-grade copper deposits already developed (Coghill et al., 2015). This trend poses a serious challenge to the mining industry, which must find ways to extract metals more efficiently and sustainably.

Unfortunately, mine depletion is not the only problem. The decline in ore grade has been accompanied by a sharp increase in energy consumption and water usage, up to 46%. In the USA, comminution accounted for 1.14% of total energy consumption in the industrial sector and 0.39% of total national consumption. In Canada, the comminution process consumed 16.7% of the total industrial sector and 1.86% of the national energy consumption. The Australian mining industry consumed 8.5% of the total national consumption in 2012 (Coffey et al., 2019). The situation is even more dramatic in South Africa. Tromans (2008) noted that the mining industry consumed about 6% (1.8% belongs to comminution) of the total national energy. According to Numbi et al. (2014), the number rocketed up to 15% with gold mines accounting for 47% of this consumption. According to Aramendia et al. (2023), the mining industry consumes 1.7% of energy annually, which is expected to increase. The relationship between decreasing ore grades and rising energy consumption has been well-documented (Jose-Luis et al., 2019; Calvo et al., 2016; Norgate and Jahanshahi, 2010; Harmsen et al., 2013). These studies highlight the need for the mining industry to adopt more energy-efficient technologies and practices to mitigate these impacts.

Comminution processes are particularly energy-intensive. In the case of challenging ore scenarios, along with being the largest energy consumer in mineral processing, the comminution is also responsible for the largest capital expenses and operating costs. According to Curry et al. (2014), mineral processes are responsible for 43–45% of the total energy consumption in mining operations, with comminution accounting for 60% of total energy expenditures (Sayadi et al., 2014). Therefore, research has always been conducted to increase efficiency in the comminution processes (Fuerstenau and Abouzeid, 2002; Tromans, 2008).

Comminution is a physical pre-treatment stage that includes crushing in the coarse range and grinding in the fine range. It aims to liberate and reduce the size of the ore. In the comminution process, autogenous (AG) and semi-autogenous grinding mills (SAG) are the dominant methods, and SAG is responsible for the most energy consumption (Avalos et al., 2020).

A SAG mill is a critical component in the mineral processing industry, employed in the comminution circuit to facilitate the liberation of valuable minerals through size reduction. SAG mills are characterised by their reliance on both the ore itself and external grinding media (typically steel balls) to achieve this reduction. The semi-autogenous nature of these mills offers operational flexibility and efficiency, making them an essential part of modern mineral processing plants.

The efficiency of a SAG mill is significantly influenced by the stages of ore preparation that precede it, namely blasting and crushing. Effective blasting at the mine site is crucial in determining the particle size distribution of the material fed into the mill (Parra et al., 2014). The effectiveness of blasting can be quantified using the F80 measure, which represents the screen size through which 80% of the material passes. Adequate fragmentation achieved during blasting reduces the need for excessive energy expenditure in subsequent processing stages, including crushing and grinding (Murr et al., 2015). Following blasting, the ore is transported to the crushing stage, further broken down into smaller, more uniform sizes. The purpose of crushing is to ensure that the particle size distribution is suitable for efficient processing in the SAG mill. The effectiveness of the crushing stage is a determinant of the mill's overall performance, as it directly impacts the feed size and the mill's grinding efficiency.

Once the ore has been appropriately sized through blasting and crushing, it is conveyed to the SAG mill. The SAG mill consists of a large rotating cylindrical drum that is partially filled with ore and steel grinding media. As the drum rotates around its horizontal axis, the material inside is lifted along the inner walls due to centrifugal forces until gravity causes it to fall. This falling motion creates a powerful impact force, the primary mechanism for breaking down the ore. Additionally, attrition forces – resulting from the friction between ore particles and grinding media – further contribute to the reduction process.

The semi-autogenous nature of the mill derives from the dual role of the ore: it serves both as the material being ground and as part of the grinding media itself. This self-grinding property, combined with the external steel balls, enhances the efficiency of the grinding process, allowing SAG mills to process a wide range of ore types and sizes without the need for pre-crushing.

The performance of a SAG mill is dependent on several critical operational variables, which must be carefully managed to ensure optimal grinding efficiency:

1. Power consumption. Power usage in a SAG mill is directly influenced by the hardness of the ore and the effectiveness of the preceding blasting and crushing stages. Harder ores generally require more energy to grind, making power consumption a key indicator of mill performance and overall efficiency.
2. Pressure and rotation. The pressure within the mill, along with its rotational speed, affects the mechanical forces applied to the ore and grinding media. These parameters influence how the material is lifted and dropped, thereby impacting the overall grinding efficiency and the amount of energy consumed.
3. Throughput. Throughput refers to the volume of material that passes through the mill over a specified period. It is a crucial metric for assessing the operational efficiency of the SAG mill, as higher throughput generally indicates more efficient processing and optimal use of the mill's capacity.

The SAG mill provides several advantages within the comminution circuit. One of its primary benefits is its ability to process large, coarse feed material directly from the mine, reducing the need for additional pre-crushing equipment. This simplifies the overall process flow and decreases operational costs. Furthermore, SAG mills are capable of handling a wide variety of ore types, making them highly adaptable in diverse mineral processing environments.

Since the SAG process is a main energy consumer, energy consumption prediction has always been a topic of research interest. Various works, including empirical and theoretical models, have been presented in the literature. The proposed works are mainly based on the pressure, sizing, water level, hardness of the feed, and charge level of the SAG mills. For instance, Curry et al. (2014) provided approaches to improve efficiency rates, including pre-concentration, coarse particle separation, and other efficient grinding technologies. These suggestions, if implemented, could lead to significant cost savings and improved environmental performance. Jnr and Morrell (1995) proposed a dynamic model that considers feeding rate, water content, recycling, feeding material size distribution, and rock hardness. This model offers a comprehensive approach to understanding the factors influencing SAG mill performance. Morrell (2004) highlighted that essential variables for prediction include breakage rate of size fractions, required breakage energy, size distribution, and slurry transportation. These factors predict throughput, size reduction, and power response of the mill. Salazar et al. (2009) investigated SAG modelling, concluding that the model effectively accounts for changes in mill velocity, feeding rate, and water flow. The liner efficiency and performance reliability are also areas of interest, knowing that the replacement of liners contributes to operational downtime and maintenance costs; thus, optimising liner design is critical for maximising mill efficiency and longevity (Yahyaei et al., 2015). Last but not least, unmeasurable variables like feeding particle size distribution and mineral composition require additional model recalibration.

Given the complexity of the factors influencing energy consumption, advanced modelling techniques have emerged as powerful tools for accurate predictions and optimisations in comminution operations. In the last decade, machine learning methods have been increasingly applied to forecast energy consumption and optimise mineral processing operations. Techniques such as neural networks and support vector machines (Curilem et al., 2011), neural-genetic algorithms (Sadat et al., 2018), recurrent neural networks (Inapakurthi et al., 2020), and deep learning models (Avalos et al., 2020) have demonstrated high accuracy and an ability to handle real-world data effectively.

In this study, the alternative strategy for understanding the relationships was employed through Bayesian and RF methods rather than solely focusing on energy consumption forecasting or fine-tuning parameters to achieve the highest prediction accuracy. The Bayesian approach incorporates uncertainty, quantifying its value and range within the model, and providing this information as part of the posterior distribution. This enhances the model's interpretability and supports more informed decision-making.

Bayesian methods provide several advantages over traditional statistical approaches. They allow for the incorporation of prior knowledge, which can be particularly useful in mining, where historical data and expert knowledge can provide valuable context. Additionally, Bayesian methods provide results as probability distributions rather than point estimates, offering a more nuanced view of potential outcomes. This probabilistic framework can be particularly valuable in decision-making processes, where understanding the range of possible outcomes and their probabilities can inform more robust strategies.

The second model, Random Forest (RF), is a machine learning method. It can be justified on several grounds. RF handles many features without dimensionality reduction, is robust against overfitting, and makes no assumptions about the functional form of the input-output relationship, making it suitable for complex, non-linear data. The versatility and robustness of RF in handling diverse datasets are particularly advantageous in mining and mineral processing, where data can be highly variable and noisy.

Additionally, RF excels at identifying essential features and making accurate predictions, but it is fundamentally a correlation-based method that does not establish causal relationships. This limitation means that while RF can identify patterns and correlations in the data, it cannot definitively determine the underlying causes of those patterns. However, Bayesian methods can address this limitation by incorporating prior knowledge and providing results as probability distributions, enhancing interpretability and understanding of the underlying data dynamics. Bayesian methods offer a framework for probabilistic inference, effectively handling complex, uncertain, or incomplete data.

The originality of this work lies in predicting energy consumption and quantifying the relationships between key variables in mining operations. The case study focuses on energy consumption predictions for SAG mills, comparing the Bayesian approach with the machine learning method. While machine learning has been widely used over the past decade, integrating Bayesian techniques introduces a novel perspective that enhances the predictions’ accuracy and interpretability. This integration is expected to provide significant insights into the dynamics of SAG mill energy consumption and contribute to developing more efficient and sustainable mining practices.

This study is structured into (1) an Introduction section, (2) a Methodology part with an explanation of the applied methods, (3) a comparison of the performance of Bayesian methods with RF in the Case study section, and (4) a Discussion of and (5) Conclusions based on the findings. This structured approach ensures that the study systematically addresses the key research questions and provides a comprehensive analysis of the predictive models.

## Methodology

In this research, energy consumption prediction for SAG mills uses two distinct methodologies: Bayesian statistics and RF. Each method offers unique advantages and insights, and their comparative analysis provides a comprehensive understanding of their effectiveness in predicting SAG mill energy consumption. This section details the methodology employed for both approaches, including the Bayesian Generalised Model (GLM) and RF, and introduces the relevance of these methods within the context of SAG mill energy consumption.

### *Bayesian methodology*

Bayesian GLM is a powerful tool for modelling and understanding complex data relationships, and it could also be used in prediction. This method has gained prominence due to advancements in computational capabilities, which have expanded its applicability across various fields, including econometrics, biology, psychology, and geology (Capretto et al., 2020; Niu et al., 2024). Bayesian methods offer flexibility in handling uncertainties and incorporating prior knowledge, making them particularly useful for modelling energy consumption in SAG mills. The Bayesian view or Bayesian statistics makes Bayesian GLM more advanced than linear regression.

#### *Bayesian statistical framework*

Bayesian statistics is founded on two core concepts: the redistribution of credibility among different possibilities and the incorporation of these possibilities into mathematical models (Kruschke, 2014). The Bayesian approach to data analysis involves several critical steps:

1. Data analysis: Preliminary exploration and understanding of the dataset, including its structure, distribution, and underlying patterns.
2. Defining the descriptive model: Formulating a model that best represents the data and captures the relationships between variables.
3. Specifying the prior distribution: Establishing prior beliefs about the parameters before observing the data. Priors can be flat, indicating a lack of specific information, or they can incorporate domain knowledge and hypotheses.
4. Applying Bayesian inference: Updating the prior distribution based on observed data to produce a posterior distribution that reflects the updated knowledge.
5. Evaluating the posterior distribution: Assessing how well the posterior distribution represents the real dataset and its accuracy in predicting outcomes.

The three main steps in Bayesian statistics involve choosing a prior distribution, a likelihood function, and a posterior distribution. Prior distribution p(*θ*) reflects the knowledge of the parameter θ before data is observed. Priors can be uniform or informative based on prior knowledge. Priors might be flat, where prior values do not contain any information, or they can hold a particular adjustment and hypothesis. The likelihood function represents the probability of the observed data given the parameters. It is the expression of the plausibility of the data given parameters p(*D|θ*). Posterior distribution combines the prior and likelihood to provide an updated distribution of the parameter after considering the data. Posterior distribution p(*θ|D*) is the result of the first two steps of the Bayesian analysis and reflects the knowledge given by our data and model. The constructed distribution reflects and considers both prior and the likelihood functions. The mathematical Equation of Bayes’ theorem is shown in the equation below:

$$p(\theta |D)=\;\frac{\;p(D|\theta )p(\theta )}{\;p(D)}$$

#### *Bayesian linear regression*

The linear regression in the frequentist view can be written mathematically as follows:

(1) Y=Xβ+ε

where Y is the dependent variable, X is the independent variable, *β* is the coefficient of the model, and the error term ε, which is assumed to be normally distributed. The best-case scenario is accepted in case of a minimal value of the error function by adjusting the *β* coefficient.

The Bayesian framework calculates linear regression in terms of probability distributions. The equation (1) can be rewritten as follows:

(2) $$Y=N(X\beta ,\;\sigma^{2})$$

Dependent variable Y is seen as a random variable that follows a Normal distribution. The mean of this distribution is given by the linear predictor, with variance $\sigma^{2}$ . Although this model is similar to the traditional frequentist model, the Bayesian view offers two significant benefits:

1. Priors or prior knowledge. Prior knowledge can be assigned to the model. If there is a high chance that variance is low, then this information could be given, and more probability mass on low values could be set as a prior.
2. Uncertainty quantification. Unlike a single deterministic coefficient *β* in linear regression, Bayesian linear regression gives the distribution and plausibility of the *β* values. If a dataset is sparse, then accordingly, *β* would be high and get a wide posterior distribution.

Linear regression assumes that the response variable is continuous and normally distributed, and a linear relationship exists between the predictors and the response. However, in many real-world situations, these assumptions do not hold. For example, binary outcomes, count data, or outcomes with skewed distributions cannot be appropriately modelled using linear regression. Therefore, the GLM is used to extend linear regression by allowing the response variable to follow any distribution from the exponential family (such as binomial, Poisson, or gamma) and by incorporating a link function that connects the predictors to the mean of the response variable in a non-linear way. This makes GLM ideal for cases where linear regression is unsuitable, such as logistic regression for binary outcomes or Poisson regression for count data.

In the Bayesian context, GLM offers further advantages by allowing the incorporation of prior knowledge about the model parameters through the use of priors. This is particularly useful in domains where domain expertise or prior research suggests that certain parameter values are more plausible than others. Additionally, Bayesian GLM provides full posterior distributions for parameters rather than point estimates, allowing for a more nuanced understanding of uncertainty. This is especially helpful when dealing with limited or noisy data, as the posterior distributions quantify the range of likely parameter values and their associated uncertainty. This ability to handle non-normal data distributions, non-linear relationships, and uncertainty makes Bayesian GLM a more robust choice than traditional linear regression in many applied settings.

Mathematically, Bayesian GLM can be written as:

*α*∼ prior *α*

*β*∼ prior *β*

*θ*∼ prior *θ*

(3) μ=a+β

(4) Y=ϕ(f(μ),θ)

where *α* is an intercept, *β* is a slope, *θ* represents additional parameters, ϕ is a distribution function, and *f* is an inverse link function.

The distribution function *ϕ* is an arbitrary distribution, it might be Normal, Gamma, Student's t or any other chosen one. *θ* represents the auxiliary parameter the distribution might obtain; in the Normal distribution, the auxiliary parameter is *σ.* Parameter *f* is an inverse link function. In the case of the Normal distribution, *f* is the identity function. The necessity of the inverse link function lies in securing the different domains’ results. For instance, if *µ* is assigned for the Negative Binomial but defined for the positive results, in this example, the transform of *µ* is required.

#### *Markov chain Monte Carlo*

The Markov Chain Monte Carlo algorithm is used in the PyMC probabilistic programming library for Python to fit the built models. Gamerman (1997) proposed the method of sampling from the posterior distribution. Since then, MCMC methods have become the core of Bayesian models in PyMC for sampling from posterior distributions in Bayesian statistics, especially when analytical solutions are infeasible. MCMC enables us to approximate complex posterior distributions by generating samples from high-probability regions. The core of MCMC is its ability to efficiently explore the parameter space and provide credible inferences about the parameters of interest.

MCMC methods are fundamental in Bayesian statistics and probabilistic programming. They provide a robust framework for handling complex, high-dimensional data and models and are essential for extracting reliable predictions from Bayesian models.

Due to its crucial importance, MCMC is called a universal inference engine. MCMC methods are the main core of Bayesian statistics and probabilistic programming by themselves. Monte Carlo arises for random numbers. A Markov chain or Markov process is a stochastic model describing the sequence of states or possible events and a set of transition probabilities. A chain is Markovian if the probability of the change of one probability depends only on the current state. In Bayesian statistics, the Markov chain is required to study the properties of MCMC samplers. The most popular MCMC method is the Metropolis–Hastings algorithm, which is applied in this work and explained in the following section.

A Markov chain is a sequence of random variables in which the future state depends only on the current state and not on the past states. A Markov chain is described by the transition probability matrix *P*, where *P(i, j)* is the probability of moving from state *i* to state *j*.

The Monte Carlo Method refers to using random sampling to obtain numerical results. In MCMC, Monte Carlo methods are used to generate samples from the posterior distribution based on the Markov chain's transitions.

The Metropolis–Hastings Algorithm is one of the most popular MCMC algorithms. It generates samples from a target distribution *p(θ|Y)* by constructing a Markov chain whose stationary distribution is the target distribution.

The algorithm involves the following steps:

1. Initialization: Start with an initial value $\theta_{0}$ .
2. Proposal:. Generate a candidate θ* from a proposal distribution $q(\theta *|\theta_{t-1})$
3. Acceptance: Calculate the acceptance ratio:
(5) $$\alpha =\min\left(1,\frac{\;p(\theta^{*}|Y)\cdot q(\theta_{t-1}|\theta^{*})}{\;p(\theta_{t-1}|Y)\cdot q(\theta^{*}|\theta_{t-1})}\right)$$
  Where p(θ*|Y) is the target distribution, and *q* is the proposal distribution.
4. Update: Accept θ* with probability α; otherwise, retain $\theta_{t-1}$
5. Repeat: Continue the process to generate a sequence of samples.

MCMC methods are versatile and can handle high-dimensional parameter spaces. They are particularly useful in Bayesian analysis for estimating complex models where closed-form solutions are impractical. MCMC's flexibility allows it to be applied across various domains, including mineral processing, which helps refine predictions of energy consumption in SAG mills.2.2 Random Forest.

The RF algorithm, introduced by Breiman (2001), has proven to be a powerful method for regression tasks where the goal is to predict continuous target variables. The RF regression model constructs an ensemble of decision trees, each trained on a bootstrapped sample of the data, and aggregates their predictions by averaging. This methodology enhances model accuracy and reduces the risk of overfitting.

In the RF regression approach, each tree is trained on a bootstrapped sample of the data. For a dataset D, with *n* instances, the RF algorithm generates multiple bootstrapped datasets D_1_, D_2_, …, D_N_ by sampling with replacement. Each dataset is used to construct a decision tree. A bootstrapped sample is drawn by randomly sampling from the original dataset with replacement, ensuring that each tree sees a unique version of the data. This process ensures variability between the trees, which improves the model's robustness.

Once a tree is constructed from the bootstrapped sample, it splits the data at each node based on the mean squared error (MSE) criterion. The MSE at each node is calculated as:

(6) $$MSE=\frac{1}{n}\overset{n}{\underset{i=1}{\sum }}\;(y_{i}-\hat{y}{)}^{2}$$

where $y_{i}$ represents the actual target value, and $\hat{y}$ is the predicted mean value at the node. The algorithm chooses the split that minimises the MSE, creating more homogeneous groups of data points within each node. This splitting process continues recursively until the stopping criteria, such as the minimum number of samples in a leaf node or maximum tree depth, are met (Biau and Scornet, 2016).

A defining feature of RF in both regression and classification is the random feature selection at each split. For regression tasks, instead of considering all the features, the algorithm selects a random subset of features at each node. This is done to reduce the correlation between trees and improve the diversity of the ensemble. The number of features to consider at each split, denoted as *m*, is typically set to *m* *=* *p/3*

The tree splits are determined by minimising the MSE within the chosen subset of features, ensuring that each tree focuses on slightly different aspects of the data. This approach reduces the risk of overfitting, which is a common issue when individual trees are grown to their full depth (Biau and Scornet, 2016).

Once all trees in the forest have been constructed, the RF regression model makes predictions by averaging the outputs of the individual trees. Given a new input, each tree provides a prediction, and the final prediction is computed as:

(7) $$\hat{y}=\frac{1}{N}\overset{N}{\underset{i=1}{\sum }}\;{\hat{y}}_{i}$$

where ${\hat{y}}_{i}$ is the prediction from the *i*-th tree, and *N* is the total number of trees in the forest. This averaging process reduces the variance of the model, making it more stable and reliable for unseen data (Breiman, 2001).

The performance of an RF regression model is highly dependent on several key hyperparameters, which play a crucial role in influencing the behaviour and predictive power of the model. In this study, several key hyperparameters were carefully selected and tuned to optimise model performance:

Number of trees. Increasing the number of trees in the forest improves model performance, but with diminishing returns after a certain point. More trees also increase the computational cost.
Maximum depth of trees. Controlling the maximum depth of the trees prevents them from becoming too specific to the training data, thereby reducing overfitting.
3. Number of features at each split. As mentioned earlier, using a random subset of features at each node ensures that the trees are less correlated, promoting better generalisation. The default for regression is to use *m* *=* *p/3*, but this can be adjusted based on the dataset size and feature interactions.
Minimum samples per leaf and split: These parameters control the minimum number of samples required to split a node or remain in a leaf node. Values of 4 and 1 were used, respectively, allowing the model to capture granular splits without overfitting.
Bootstrap Sampling: This defines whether the model uses bootstrap samples to build individual trees. Bootstrap sampling was disabled to enhance efficiency and focus on deterministic splits.

The hyperparameters were tuned using grid search and cross-validation techniques. Grid search systematically evaluated combinations of hyperparameter values to find the optimal configuration, while cross-validation ensured the model generalised well to unseen data by splitting the dataset into multiple training and validation sets.

RF excels at reducing overfitting in regression tasks through its ensemble approach. Its reliance on bootstrap builds each tree from a randomly sampled subset of data. During tree construction, a random subset of features is chosen for each split, reducing correlation among trees. This approach reduces variance and increases model stability, preventing any tree from overfitting to noise in the training data.

This technique ensures that no single tree dominates the prediction process, thereby preventing overfitting noise in the training data (Biau and Scornet, 2016). Additionally, RF provides out-of-bag (OOB) error estimates, which serve as an internal validation measure without needing a separate test set.

Random Forest models are widely recognised for their robustness and versatility, performing well across diverse datasets with minimal tuning. Their ability to handle noisy data, high-dimensional inputs, and complex interactions makes them an appealing choice for predictive tasks, especially given their computational efficiency relative to other machine learning models.

Random forests, despite their strengths, have notable disadvantages. Their ‘black-box’ nature makes them difficult to interpret, hindering the understanding of how input variables influence predictions. Variable importance measures can be misleading, particularly in the presence of highly correlated features, as importance tends to be distributed among correlated variables rather than pinpointing the most influential ones. In high-dimensional settings, random forests may suffer from bias, especially when the number of informative predictors is small relative to the total variables (Biau and Scornet, 2016).

## Case study

This case study focuses on a copper concentrator plant located in Peru, where various stages of mining and milling processing are optimised to improve operational efficiency and reduce costs. The study investigates the impact of geological characteristics, blasting practices, and operational parameters on the performance of the SAG mill, which plays a critical role in the size reduction of ore to liberate valuable minerals.

The case study corresponds to the copper mining and SAG mill grinding process. The operational dataset consists of geological, blasting, and plant domain variables. Geological information includes the percentage of five rock alteration types (intrusive A, intrusive B, actinolite-tremolite, hornfels, and serpentine-magnetite), copper grade, and rock hardness obtained from laboratory test results. The five alteration types presented in the dataset refer to changes in a rock's mineral composition due to geological processes such as heat, pressure, and fluid interactions. Tremolite and actinolite share the same chemical formula and very similar minerals, which belong to the calcium amphibole group, forming a series of isomorphic mixed crystals that can substitute for each other based on chemical conditions. These minerals appear as long prismatic, radial needles or fibrous aggregates, with actinolite asbestos forming fibrous structures similar to asbestos.

Serpentine and magnetite are typically formed through the hydrothermal alteration of ultramafic rocks, a process called serpentinization. During this process, minerals like olivine and pyroxene are hydrated, resulting in the formation of serpentine, while the oxidation of iron within these minerals produces magnetite. This association indicates significant fluid-rock interactions, commonly occurring in tectonically active environments.

Hornfels is a fine-grained metamorphic rock formed through contact metamorphism, which occurs when hot magma intrudes into surrounding rocks. The intense heat from the magma alters the original rock's mineral structure without significant pressure, resulting in the formation of hornfels. This process creates a hard, dense rock, often found near the margins of intrusive bodies like sills and dikes.

The study assigns values to these alterations, summing to 100%, providing a detailed breakdown of the mineral composition. Actinolite-tremolite forms under specific metamorphic conditions, while serpentine-magnetite is linked to hydrothermal alteration. Hornfels, on the other hand, is a fine-grained metamorphic rock formed by contact metamorphism, typically occurring near intrusive bodies like magma. Intrusive_A and Intrusive_B likely refer to different stages or types of rock alteration caused by magmatic intrusions. The geological variables represent the percentage of each geological type in the studied mining block. Additionally, the rock hardness variable, determined by drill penetration rates, is vital for mining operations, as harder rocks resist drilling, impacting blasting and extraction processes.

The blasting results are represented by F80, the size threshold at 80% of the material fed to the comminution processes. Other variables include coarse (blasted materials larger than 10 cm), fines (fraction of the result less than 2.5 cm), and intermediate (material between 2.5 and 10 cm).

**Figure 1.**
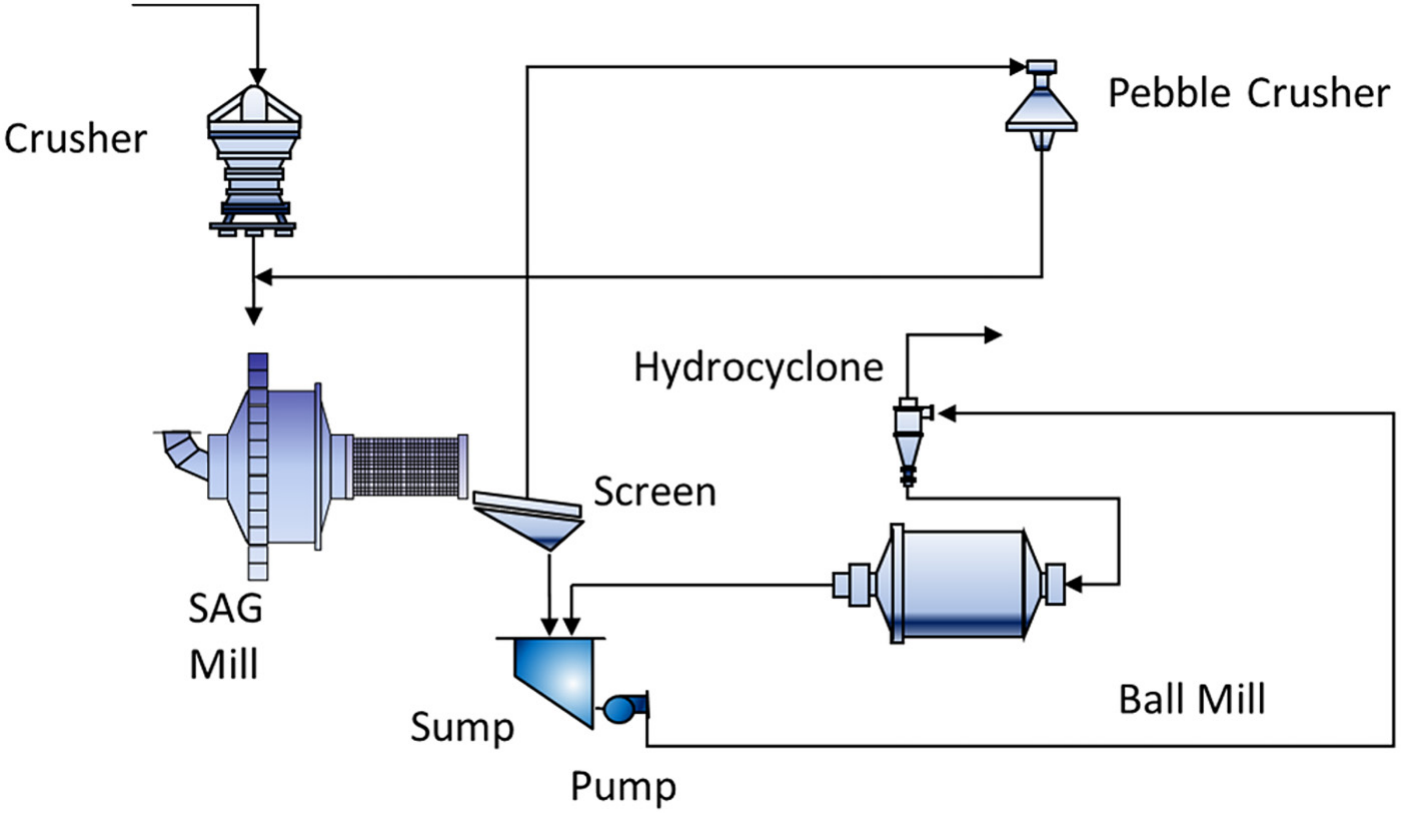
Simplified SAG process circuit (Modified from Magzumov and Kumral (2025)).

Following blasting, the ore is transported to the comminution stage, where it is broken down into smaller, more manageable sizes for grinding. The only information from the crushing stage is the product of crushed material or fresh feed (t/h). Additionally, in the grinding stage, the throughput includes recirculated pebbles and coarse fragments that were not fully ground in the initial pass through the mill. These pebbles are either returned to the SAG mill for further grinding or to a pebble crusher for size reduction. Key operational variables for the SAG mill include power consumption (MW), mill pressure (kPa), and rotation (RPM) of the SAG mill. The simplified grinding flow sheet is presented in Figure 1. This diagram illustrates the flow of materials and operational variables in the SAG milling process, highlighting key components such as the crusher machine, SAG mill, sump, pump, hydro cyclone, pebble crusher, and ball mill.

**Figure 2.**
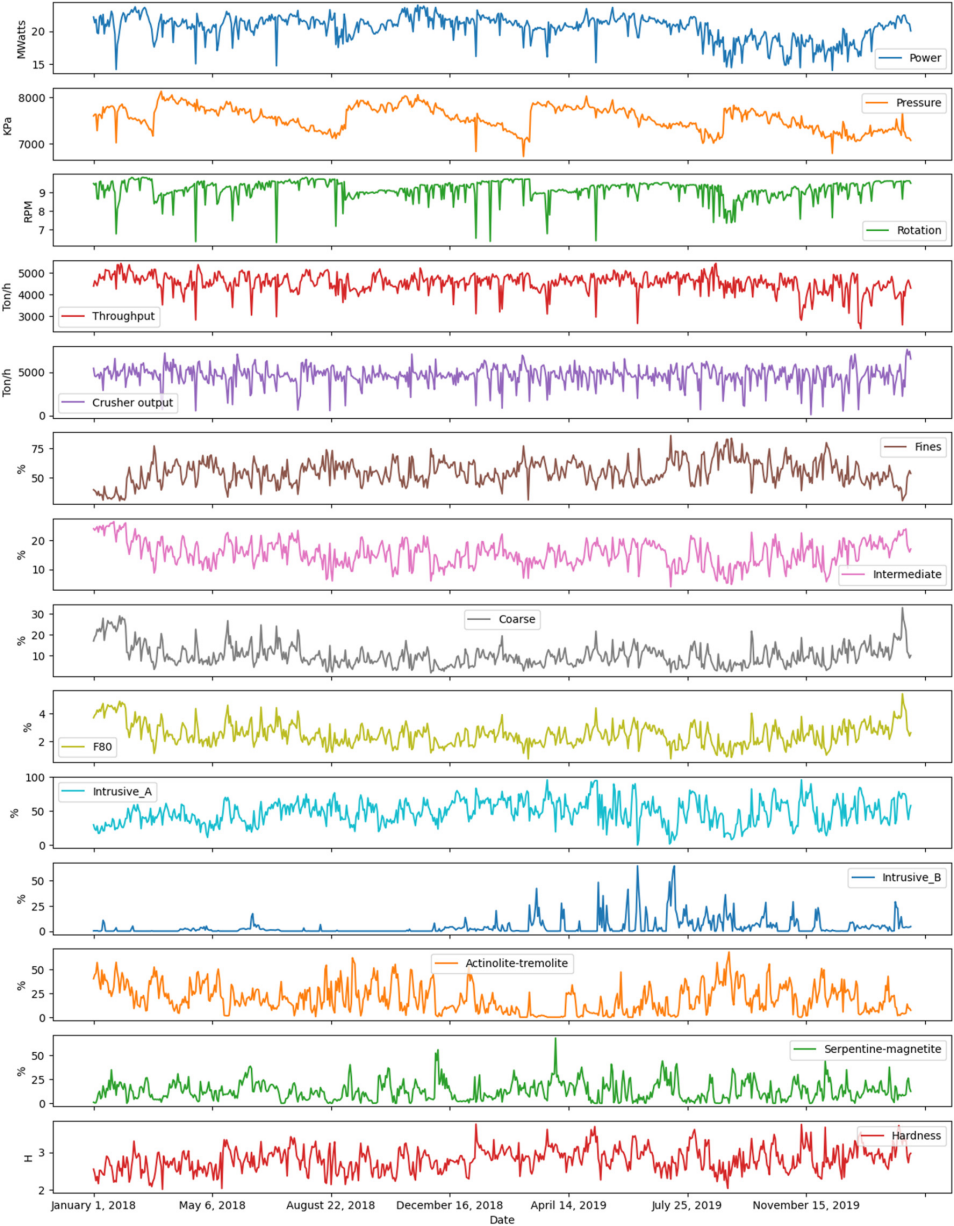
The graphical representation of the variables of interest.

Figure 2 visually presents the working dataset gathered from January 20, 2018, to February 20, 2020. The dataset consists of 686 daily operational observations. This multi-panel plot provides time series data for various operational variables, including power consumption, pressure, rotation speed, throughput, and geological compositions. It allows for an in-depth visual analysis of their trends over time. The operational variables from the mining and SAG grinding processes shown in Figure 2 are summarised in Table 1.

**Table 1.** Operational variables from the mining and SAG grinding processes.

	Unit	Lower limit	Mean value	Upper limit
F80	%	1.93	2.41	2.98
Fines	%	47.81	54.15	61.65
Intermediate	%	12.53	15.57	18.67
Coarse	%	6.41	9.09	12.56
Intrusive A	%	35.30	47.73	60.62
Intrusive B	%	0	0.32	3.93
Actinolite-Tremolite	%	7.62	16.73	28.75
Serpentine-magnetite	%	4.84	10.88	18.83
Hardness	dimensionless	2.59	2.80	3.00
Copper grade	%	0.60	0.682	0.78
SAG power Consumption	MW	19.60	21.04	21.78
SAG pressure	kPa	7360.33	7565.26	7761.63
SAG rotation	RPM	8.98	9.27	9.48
Crushed materials	t/h	4209.20	4746.76	5277.84
Throughput	t/h	4329.99	4598.37	4803.25
Liner’s campaign	Discrete variable	‐	‐	‐

**Figure 3.**
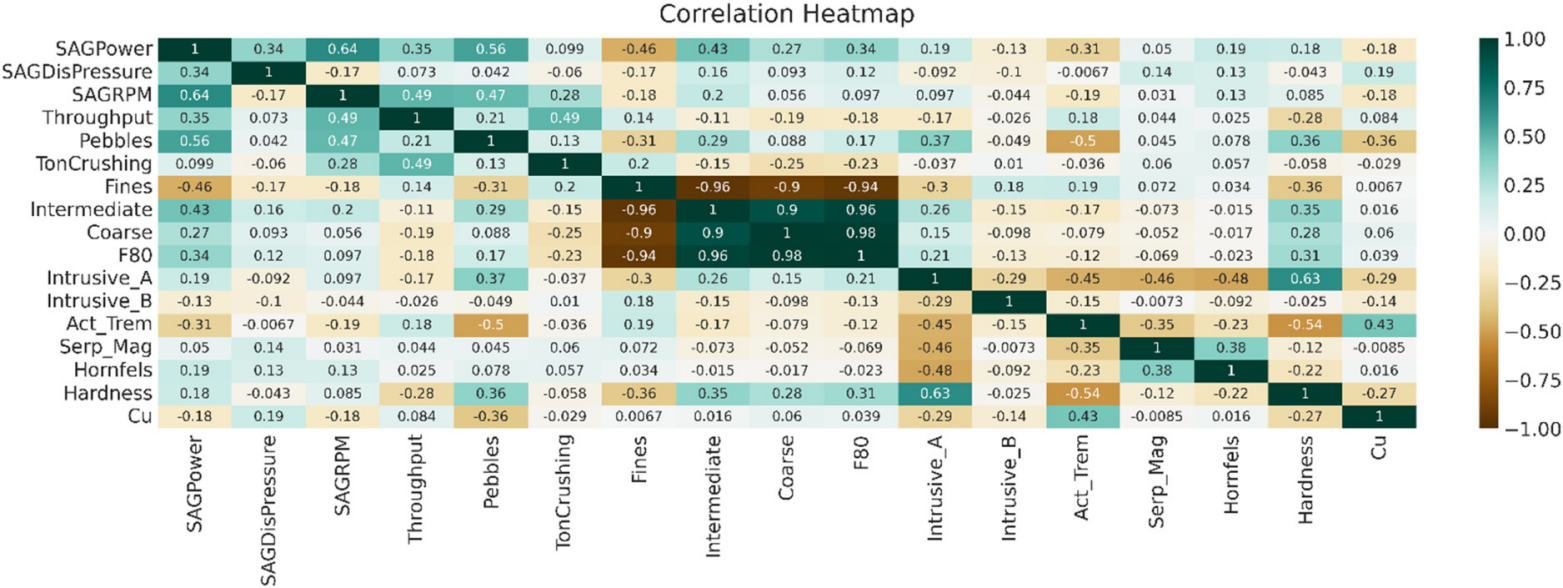
A Spearman correlation of the studied variables.

The preliminary analysis of the studied variables involved Spearman correlation to reveal the dynamics between variables. The Spearman correlation of studies is shown in Figure 3. This heatmap visualises the correlation coefficients between different variables, identifying strong positive and negative correlations that are critical for understanding the interplay between geological and operational factors. Figure 3 shows a positive correlation between blasted material fractions (coarse, intermediate, and F80) and a high negative correlation between fine particle sizes and blasted variables. Significant correlations also exist between intrusive A and hardness, and between SAG mill rotation speed and power consumption, indicating potential links between geological variables and power consumption.

**Figure 4.**
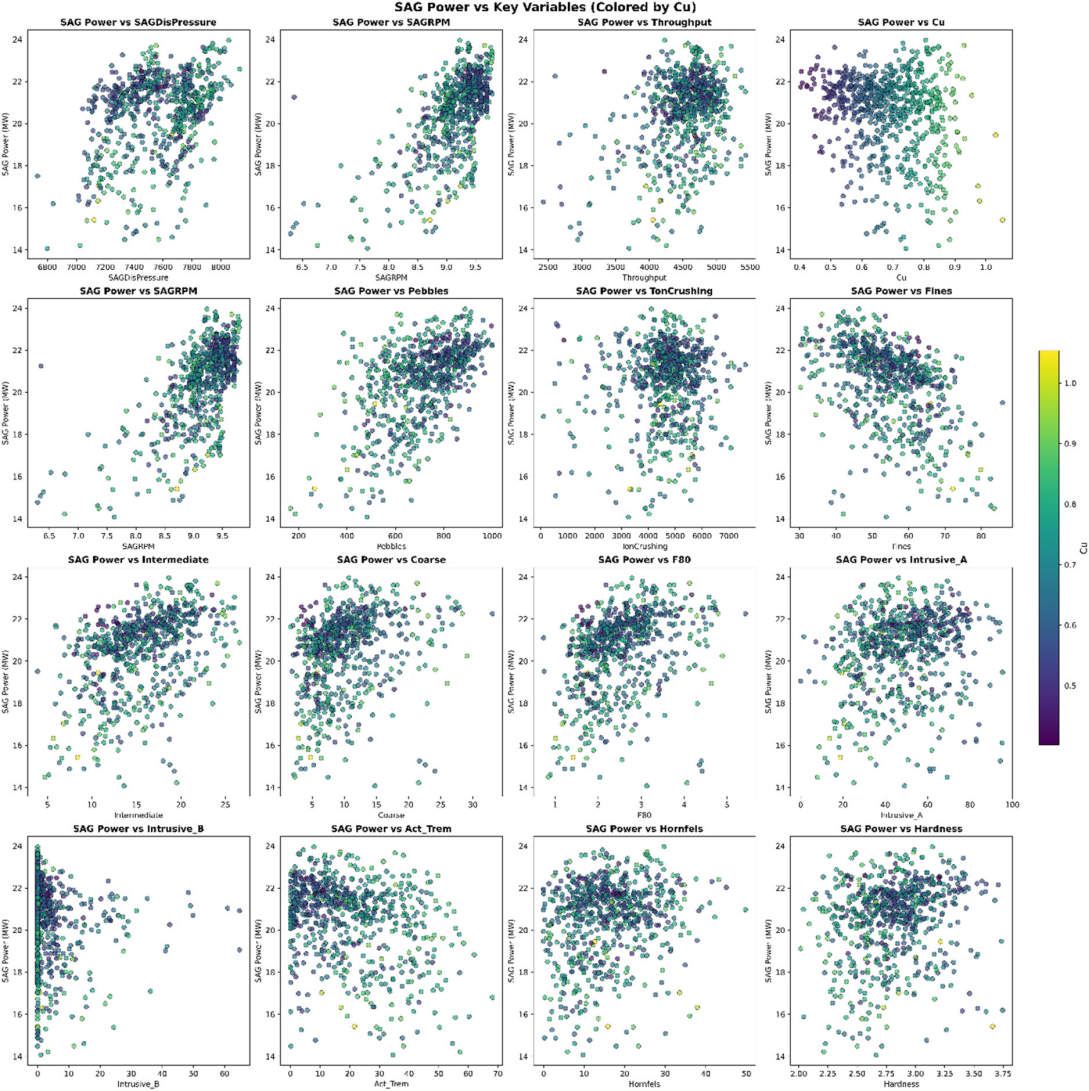
Scatter plot of the operational variables.

Further research focused on variable selection for RF and Bayesian statistics methods. As shown in Figure 4, the scatterplot matrix helps visualise relationships between variables, assisting in identifying key predictors for proposed models. The robustness of the proposed models is increased by ensuring that the selected predictors are symmetrically distributed without significant outliers. Additionally, the minimal pairwise correlation between variables suggests that multicollinearity is not a significant issue, which further enhances the reliability of the predictive models.

### *Variable selection*

To predict energy consumption accurately, the necessary variables need to be chosen. Operating with 18 variables increases the risk of overfitting, where the model might capture noise rather than trends, making it difficult to determine the role of individual variables. High variable numbers also increase computational difficulty.

Variable selection offers several benefits: (1) Reduction of measurement costs, decreasing the number of required tests, saving expenditures and increasing decision-making efficiency by reducing operational time. (2) Reduction of computational cost, as operating with fewer variables reduces excessive computational demands. (3) Understanding correlation structures and finding variables that facilitate better predictions.

Different methods can implement variable selection. In this research, projection predictive inference was applied (Piironen et al., 2020; McLatchie et al., 2023). The main steps include:

1. Providing a reference model with available variables.
2. Building several submodels.
3. Generating the posterior distribution of the reference model into the submodels.
4. Comparing the most concise models with the reference model and choosing the nearest in terms of accuracy.

To reduce measurement costs, the number of variables was decreased. Additionally, the results from Magzumov and Kumral (2025), incorporating Granger causality and Toda-Yamamoto causality tests, were considered. Due to the high correlation of blasting results, an intermediate variable was selected, with hardness chosen from the geological domain, along with copper grade. For plant variables, throughput and SAG mill rotation were included to study the energy consumption of the SAG mill.

The geological variables (Intrusive_A, Intrusive_B, Actinolite-Tremolite, Serpentine-Magnetite, and Hornfels) collectively sum to 100%, representing the mineralogical composition. Among these, Intrusive_B and Serpentine-Magnetite exhibit significantly lower average and median values (3.9% and 12.8% for the average, 0.3% and 10.8% for the median) compared to other variables. Furthermore, these two variables contain a substantial proportion of zeros, complicating their integration into statistical models and potentially impairing model convergence and stability. Excluding Campaign, a categorical variable, the remaining 15 operational variables were subjected to rigorous analysis using the ‘Kulprit’ package, which employs projection predictive inference. This approach identifies the optimal subset of variables, balancing predictive accuracy and interpretability without sacrificing critical information. By focusing on variables that meaningfully contribute to model performance, this study ensures robust and computationally efficient modelling.

As the initial 18 variables were reduced to 9, Kulprit's projection predictive inference in Python was applied for feature selection. ‘Kulprit, a Python package for feature selection, was used to identify critical predictors, optimising the projection predictive inference model’.

After defining the Bayesian model, Kulprit was used to compute the Expected Log Predictive Density (ELPD), a metric in Bayesian statistics that evaluates the predictive accuracy of models. The ELPD metric helps assess the quality of the Bayesian model predictions when forecasting SAG mill energy consumption, ensuring that the model can generalise effectively to new datasets. The projection predictive inference method is then applied to generate submodels by selecting the most relevant parameters. These submodels are created to match the predictive performance of the more complex reference model, ensuring that the reduced model retains similar accuracy while being more interpretable and computationally efficient.

The initial stage is to define the Bayesian model, and Kulprit computes the ELPD. Therefore, the log likelihood is required to perform the analysis. The ELPD is a measure used in statistics to evaluate the predictive accuracy of a model. It is commonly used in the context of Bayesian statistics and model comparison. The ELPD is essentially the sum of the log predictive densities of a model for a set of data points, providing an estimate of how well the model is expected to predict new data.

After assigning the Bayesian model, Kulprit could be used. Kulprit performs a search for a submodel close to the parameters of the reference model. Kulprit compares the submodels in accordance with the ELPD, from the lowest to the highest values. The *X*-axis shows the size of the submodel, and the ELPD values are shown on the *Y*-axis. The dashed line shows a reference model value.

The models are represented as follows:

0 SAGPower ∼ 1

1 SAGPower ∼ SAGRPM

2 SAGPower ∼ SAGRPM + Intermediate

3 SAGPower ∼ SAGRPM + Intermediate + Hardness

4 SAGPower ∼ SAGRPM + Intermediate + Hardness + Throughput

5 SAGPower ∼ SAGRPM + Intermediate + Hardness + Throughput + Hornfels

6 SAGPower ∼ SAGRPM + Intermediate + Hardness + Throughput + Hornfels + F80

7 SAGPower ∼ SAGRPM + Intermediate + Hardness + Throughput + Hornfels + F80 + Cu

8 SAGPower ∼ SAGRPM + Intermediate + Hardness + Throughput + Hornfels + F80 + Cu + Act_ Trem

9 SAGPower ∼ SAGRPM + Intermediate + Hardness + Throughput + Hornfels + F80 + Cu + Act_Trem + Coarse

10 SAGPower ∼ SAGRPM + Intermediate + Hardness + Throughput + Hornfels + F80 + Cu + Act_Trem + Coarse + SAGDisPressure

11 SAGPower ∼ SAGRPM + Intermediate + Hardness + Throughput + Hornfels + F80 + Cu + Act_Trem + Coarse + SAGDisPressure + Fines

12 SAGPower ∼ SAGRPM + Intermediate + Hardness + Throughput + Hornfels + F80 + Cu + Act_Trem + Coarse + SAGDisPressure + Fines + Intrusive_A

13 SAGPower ∼ SAGRPM + Intermediate + Hardness + Throughput + Hornfels + F80 + Cu + Act_Trem + Coarse + SAGDisPressure + Fines + Intrusive_A + Pebbles

14 SAGPower ∼ SAGRPM + Intermediate + Hardness + Throughput + Hornfels + F80 + Cu + Act_Trem + Coarse + SAGDisPressure + Fines + Intrusive_A + Pebbles + TonCrushing

**Figure 5.**
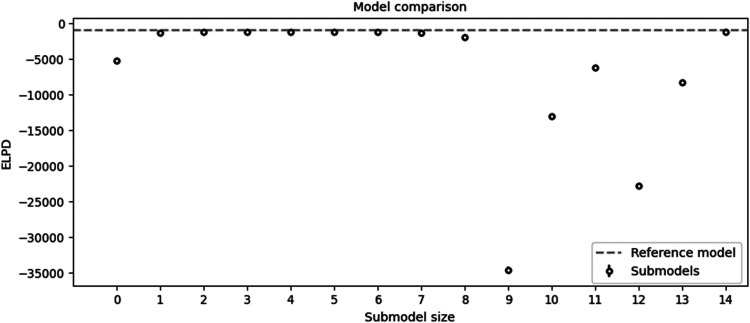
Comparison of the submodels obtained with Kulprit.

Figure 5 compares the Expected Log Predictive Density (ELPD) of various submodels against the reference model, highlighting their predictive accuracy and simplicity. Most submodels (except those with sizes 9–13) perform similarly to the reference model, as indicated by their close alignment with the dashed line. While the fifth submodel demonstrated strong alignment, I opted for the eighth submodel. This decision was made due to the minimal difference between the seventh and eighth submodels, with the additional variable in the eighth submodel offering potential benefits in capturing key predictive factors. The fourteenth submodel, while including the most variables, was computationally challenging and did not provide significant performance improvements, reinforcing the selection of the more efficient eighth submodel.

The selected variables – hardness, throughput, copper grade, and SAG RPM – were included based on their strong predictive contributions. Hardness reflects the resistance of the ore to grinding, making it a critical factor for energy consumption. Throughput directly impacts the load and energy demand of the mill. Copper grade, although negatively correlated with SAG power, provides insights into ore quality, affecting grinding efficiency. SAG RPM is a key operational variable that is directly influenced. The primary aim is to develop predictive models for power consumption. Intermediate, as a significant independent variable, facilitates understanding energy consumption interrelations. Understanding these variables can significantly improve energy consumption predictions, impacting operational strategies. Traditional literature often prioritises F80, which in this work primarily consists of fine particles (69%), with intermediate and coarse particles contributing 29% and 12%, respectively. Therefore, in this study, both Intermediate and F80 were used, although fines and coarse were not chosen by the Kulprit model.

The inclusion of variables such as Hardness, Throughput, Copper Grade, and SAG RPM aligns with their operational importance in influencing SAG mill energy consumption. Hardness, for example, directly correlates with the energy required to break the ore, while Throughput represents the volume processed, a critical factor for optimising energy efficiency. Copper Grade was selected as it influences the ore's grindability, with higher grades often requiring less energy for comminution. Similarly, SAG RPM significantly impacts the mechanical forces exerted during grinding, making it a key operational variable.

Interestingly, variables such as Intrusive_A, SAG mill discharge pressure, and pebbles were not chosen by the model, likely because their influence on energy consumption was either minimal or redundant when combined with other predictors. This highlights the model's ability to prioritise the most impactful variables while excluding less significant ones.

Additionally, the inclusion of geological factors such as Hornfels and Actinolite-Tremolite provides valuable insights into the ore's mineralogical properties. These variables offer a glimpse into the geological side of the ore, helping bridge the gap between geological composition and its influence on energy consumption during grinding.

It is important to note that projection predictive inference, which forms the basis of the Kulprit package, cannot detect causality unless the admissible set of predictors is included (McLatchie et al., 2023). However, in our case, power consumption is directly influenced by variables such as hardness and fraction size, which could potentially confound or impact the relationship between SAG mill rotation speed and SAG mill power consumption. By incorporating these variables within the GLM framework, projection predictive inference can be applied to support causal inference.

### *Regression variance*

A Bayesian GLM approach was chosen to predict SAG mill energy consumption for its robustness. Due to unknown regression coefficients, prior distributions were used. Normal distribution priors were selected, aligning with the dataset's distribution. Expert judgment was applied to avoid overfitting, emphasising domain knowledge over observed data.

**Figure 6.**
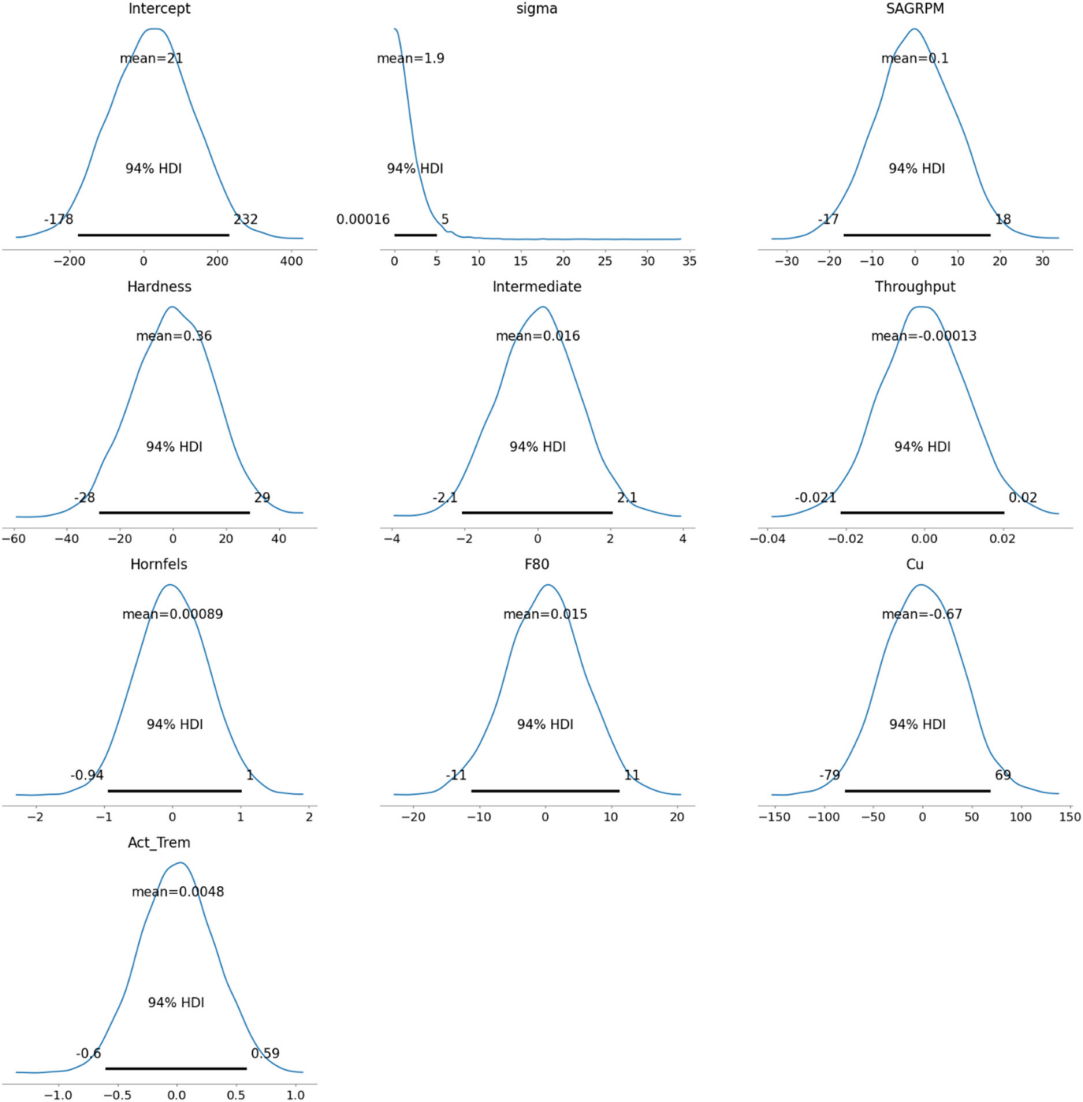
The priors of the model.

The prior distribution for all variables was chosen to exhibit a normal distribution due to its flexibility in taking any value along the real line. The real line refers to the continuous set of all real numbers, from negative infinity to positive infinity, allowing the normal distribution to cover a wide range of possible values for the variables. Since the original data distribution, as shown in Figure 4, closely resembles a normal distribution, it was decided to proceed with normal priors. It is also possible to adjust the priors to better match the real data or modify them based on expert judgment to reflect reasonable expectations for the parameters. Figure 6 presents the prior distributions for the model's parameters, all set as normal distributions, except for the auxiliary variable sigma, to align with the ‘weakly informative priors’.

As Westfall (2017) indicated, the normal distribution is commonly used to define ‘weakly informative priors’ in Bayesian models, which help stabilise the model by preventing it from exploring unreasonable or extreme parameter values. However, the sigma parameter, known as an auxiliary parameter, is treated differently. Sigma does not model the relationship between predictors and the response directly; instead, it represents the uncertainty or residual variability in the data – essentially, how much the observed data can deviate from the mean predictions. A Half-Student's *t*-distribution is often used for sigma because it ensures positivity (since standard deviation cannot be negative) and provides heavier tails, allowing for greater flexibility in handling outliers and noisy data. This makes the model more robust compared to using a normal distribution for sigma (Martin, 2024).

In Bayesian regression, a prior is placed on the intercept (as with other parameters) to incorporate prior knowledge or reasonable expectations about its value. This helps regularise the model, especially in cases with limited data, by preventing extreme or implausible estimates for the intercept based on the data alone.

Furthermore, the prior distribution includes the Highest Density Interval (HDI) values. HDI is a Bayesian credible interval that represents the range within which the true parameter value lies with a certain probability, usually 94%, but could be chosen. Unlike frequentist confidence intervals at 5%, the HDI provides the most credible values of the parameter, ensuring that every point inside the interval has a higher probability density than points outside it.

**Figure 7.**
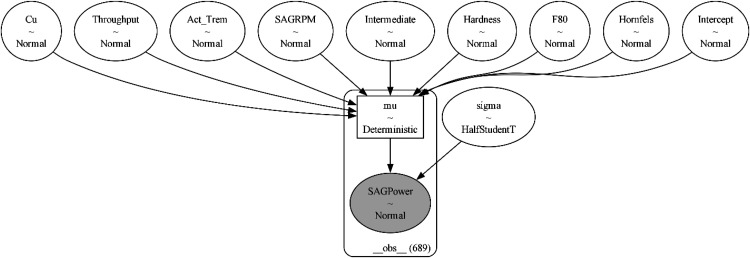
The graphical representation of the model.

Figure 7 shows the graphical representation of the model Figure 7. The model diagram illustrates the structure of the Bayesian GLM, depicting the relationships between the response variable (SAG Power) and predictors (Intermediate, Hardness, Throughput, Cu, SAGRPM). The intercept and response variable are shown as normal distributions, while the response variable's sigma variable is shown as Half-Student t due to its arbitrary nature.

**Figure 8.**
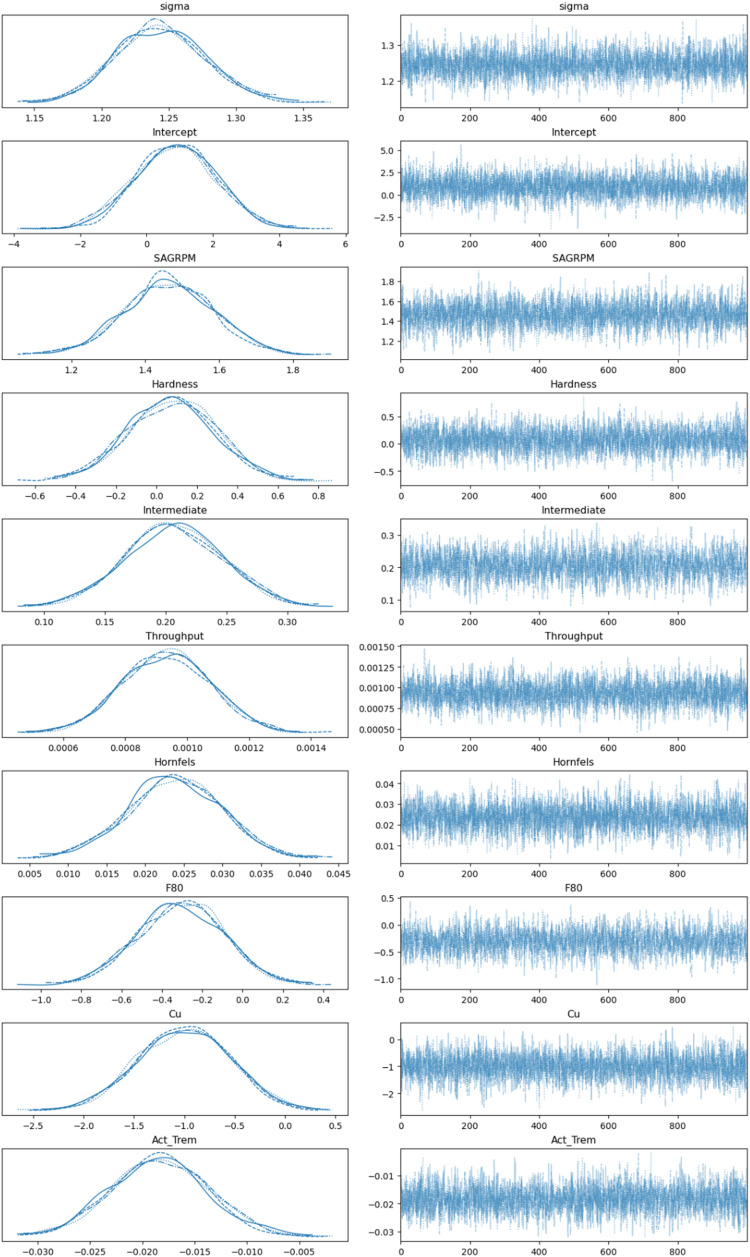
MCMC sampling.

The most crucial part of the Bayesian process is MCMC sampling, as shown in Figure 8, which efficiently explores the parameter space by generating samples that represent the posterior distribution. MCMC is indispensable for solving complex real-world cases, such as the one presented in this work. The model fitting process used MCMC simulations to estimate posterior distributions of model parameters. MCMC took 1000 samples per chain, with four chains totalling 8000 samples. The left side of the trace plot is called Kernel Density Estimation (KDE), which is a non-parametric method for estimating the probability density function of a random variable. It smooths out the observed data points to create a continuous curve that represents the distribution, making it easier to visualise the underlying distribution of the data. Since all variables have a similar curve-shaped distribution, it indicates proper sampling execution.

The individual values at each sampling step are shown on the right side of the trace plot in Figure 8. In MCMC sampling, achieving convergence across multiple chains is critical for ensuring that the samples accurately represent the target posterior distribution. Ideally, when observing trace plots, the chains should appear noisy and indistinguishable from one another, indicating that they have successfully explored the parameter space and are sampling from the same posterior distribution. This lack of discernible patterns across chains demonstrates that the sampler has overcome its initial conditions and reached a stable state, where the samples can be trusted for valid inference. Conversely, if the chains exhibit systematic differences or trends, it may suggest a lack of convergence, raising concerns about the reliability of the estimates. In the presented case, convergence was achieved successfully.

**Figure 9.**
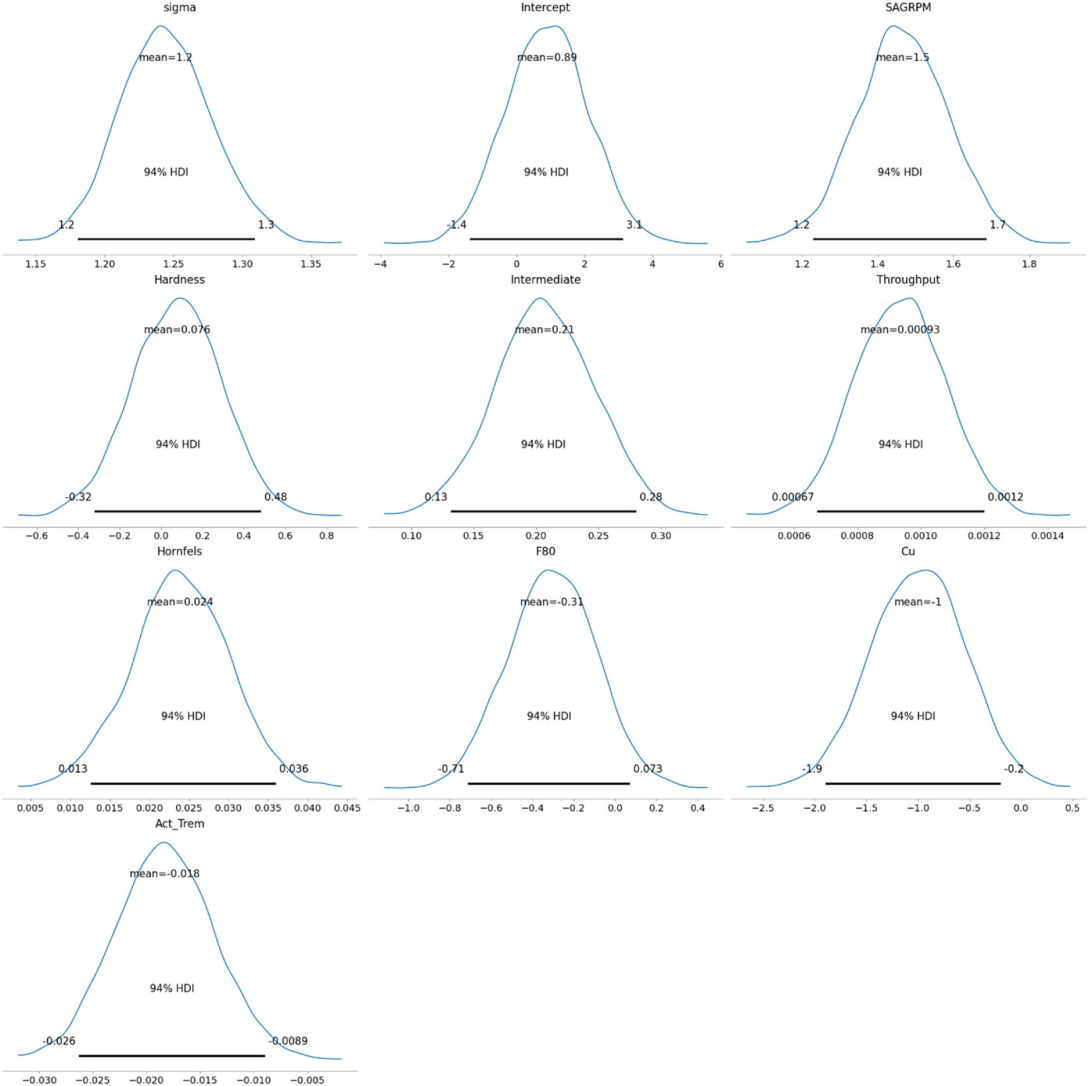
Posterior distribution of the model.

As a final part, the posterior distribution results are shown in Figure 9. It is clear how posterior distribution values have changed when prior values are faced with real data. This figure presents the posterior distributions of the model parameters, showing the mean and 94% HDI for each parameter. Bayesian GLM provides a distribution of variables, allowing for the consideration of worst- and best-case scenarios. The Bayesian GLM was constructed with a normal distribution for energy consumption (E) and a linear predictor based on collected covariates:

$$E_{i}=N(\mu ,\;\sigma^{2})$$

$$\begin{array}{rl} \mathrm{SAG}\;\mathrm{Power} & =\;\beta_{0}+\beta_{1}\mathrm{SAGRPM}+\beta_{2}\mathrm{Hardness}+\beta_{3}\mathrm{Intermediate}\\ & \;+\beta_{4}\mathrm{Throughput}+\beta_{5}\mathrm{Hornfels}+\beta_{6}F80\\ & \;+\beta_{7}\mathrm{Cu}+\beta_{8}\mathrm{Actinolite}\_\mathrm{Tremolite} \end{array}$$

If average values of the HDI are used, the Bayesian GLM coefficients’ could be shown as:

SAG Power = 0.890 + 1.465 SAGRPM + 0.076 Hardness + 0.207 Intermediate + 0.001 Throughput +0.024 Hornfels −0.306 F80 – 1.003 Cu – 0.018 Actinolite_Tremolite

However, as a risk analysis, the best- and worst-case scenarios, any coefficient values could be taken from the given posterior distributions.

To evaluate predictive performance, Leave-One-Out Cross-Validation (LOO) was employed in this study. LOO involves systematically removing one data point from the dataset, training the model on the remaining data, and testing it on the excluded point. This process is repeated for every observation, ensuring an unbiased estimate of the model's predictive capability. The evaluation metric, ELPD, quantifies the model's ability to predict unseen data, with higher ELPD values indicating better generalisation.

An integral component of LOO is the Pareto k-diagnostics, which detects influential data points that could distort the results. If all Pareto k values remain below the threshold of 0.7, the model's performance can be deemed reliable. Bayesian computational methods, such as importance sampling, further enhance the efficiency of LOO, establishing it as a robust and widely accepted standard for model evaluation in Bayesian analysis. (Martin, 2024)

The performance of the Bayesian Generalised Linear Model (GLM) was evaluated using LOO cross-validation. The analysis resulted in an ELPD of −1134.45, with a standard error of 21.82, demonstrating the model's strong predictive capability. The Pareto *k*-diagnostics confirmed that all observations fell within the acceptable range (*k*∈[−∞,0.7]), indicating that no influential data points were present to bias the results.

These findings confirm the reliability and generalisation capability of the Bayesian GLM. By providing a robust evaluation of predictive performance, LOO ensures that the model is well-suited for forecasting energy consumption in SAG mills, while maintaining interpretability and computational efficiency.

**Figure 10.**
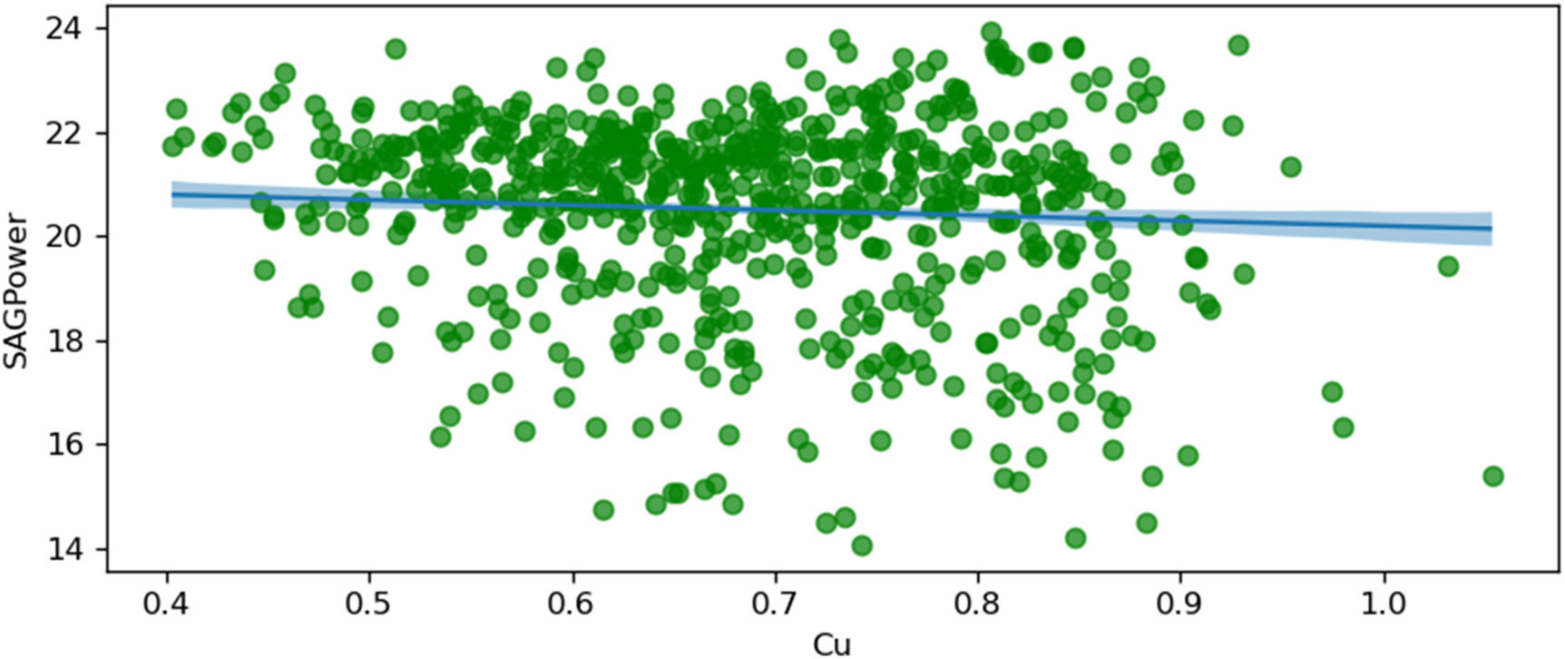
SAG power and copper grade (Cu).

**Figure 11.**
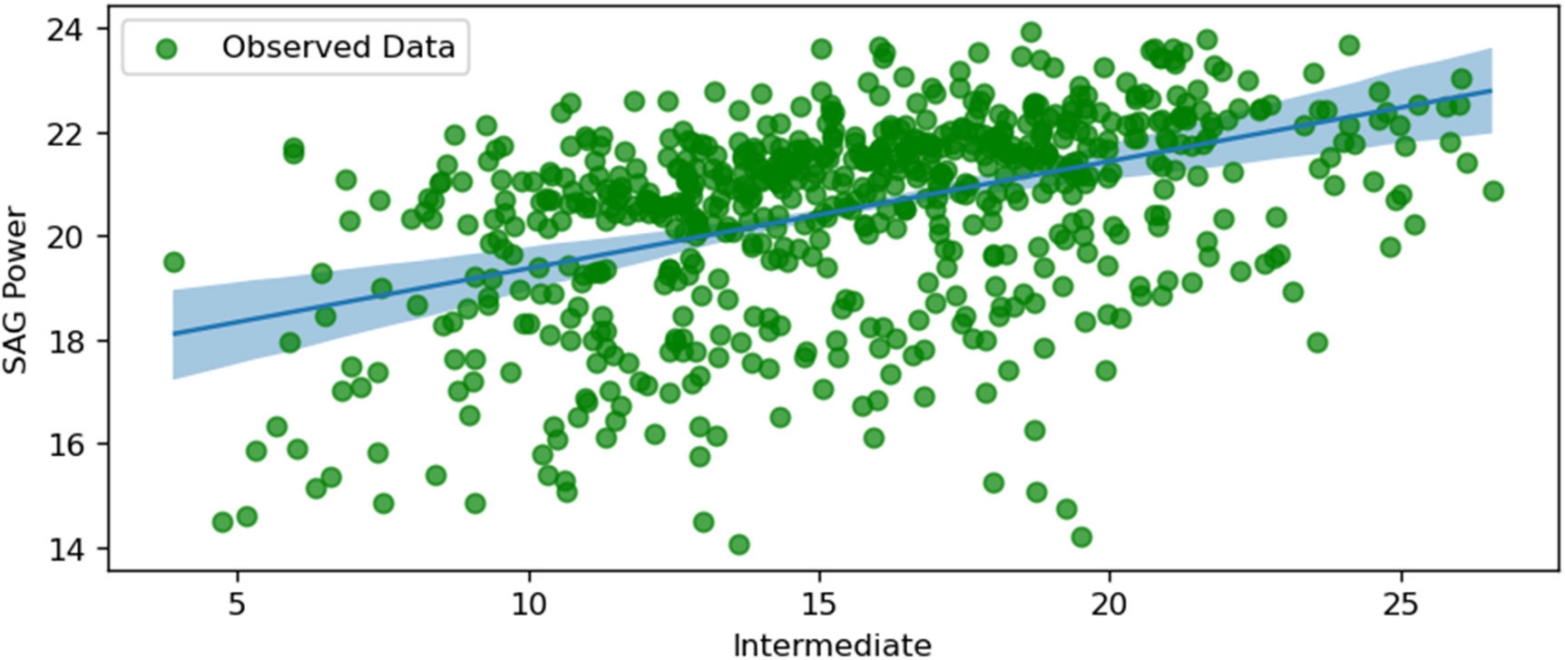
SAG power and intermediate.

**Figure 12.**
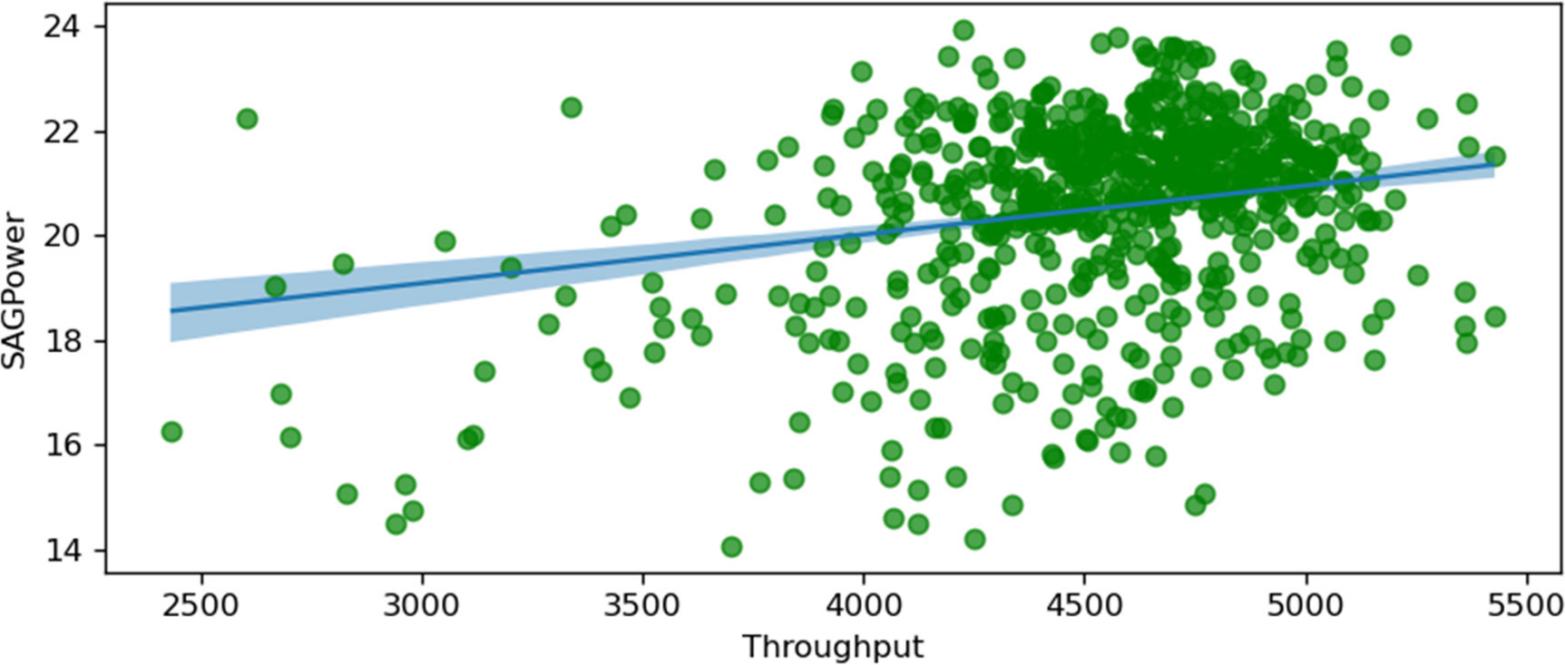
SAG power and throughput.

**Figure 13.**
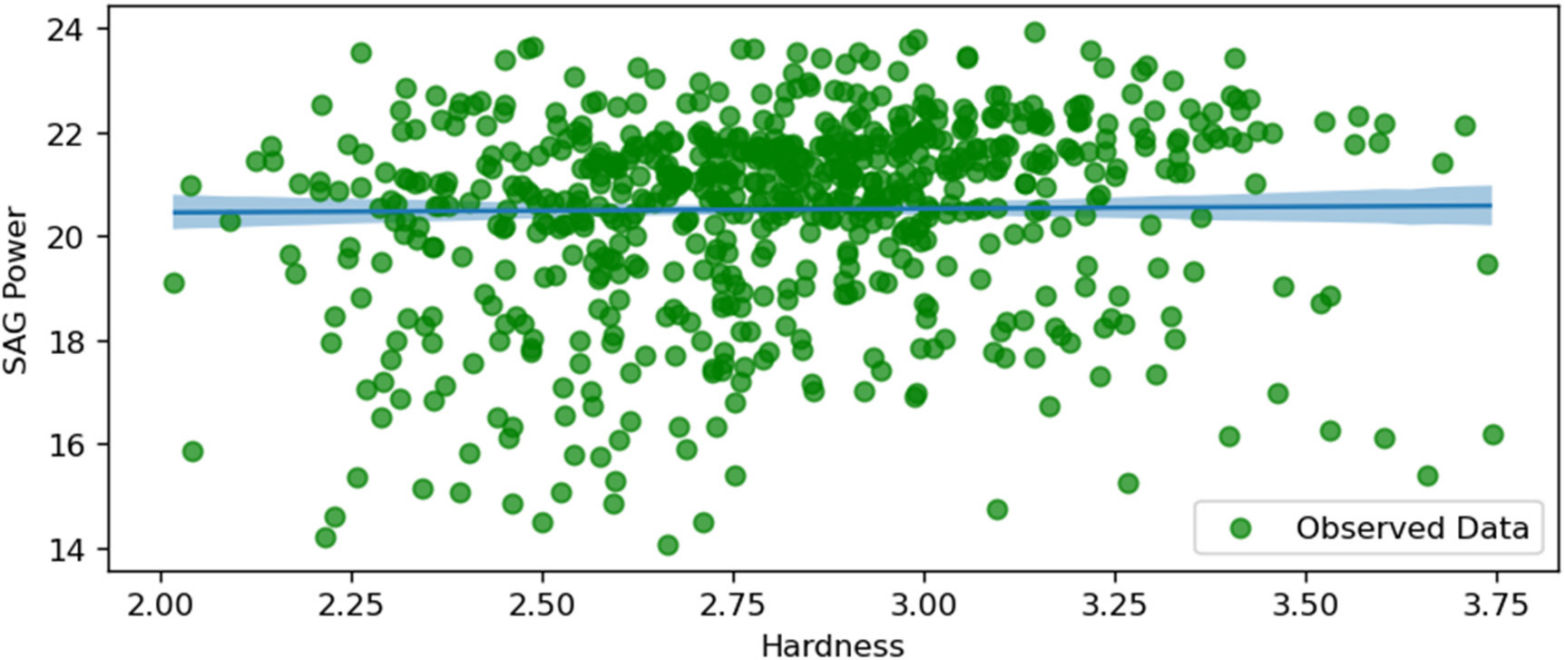
SAG power and hardness.

**Figure 14.**
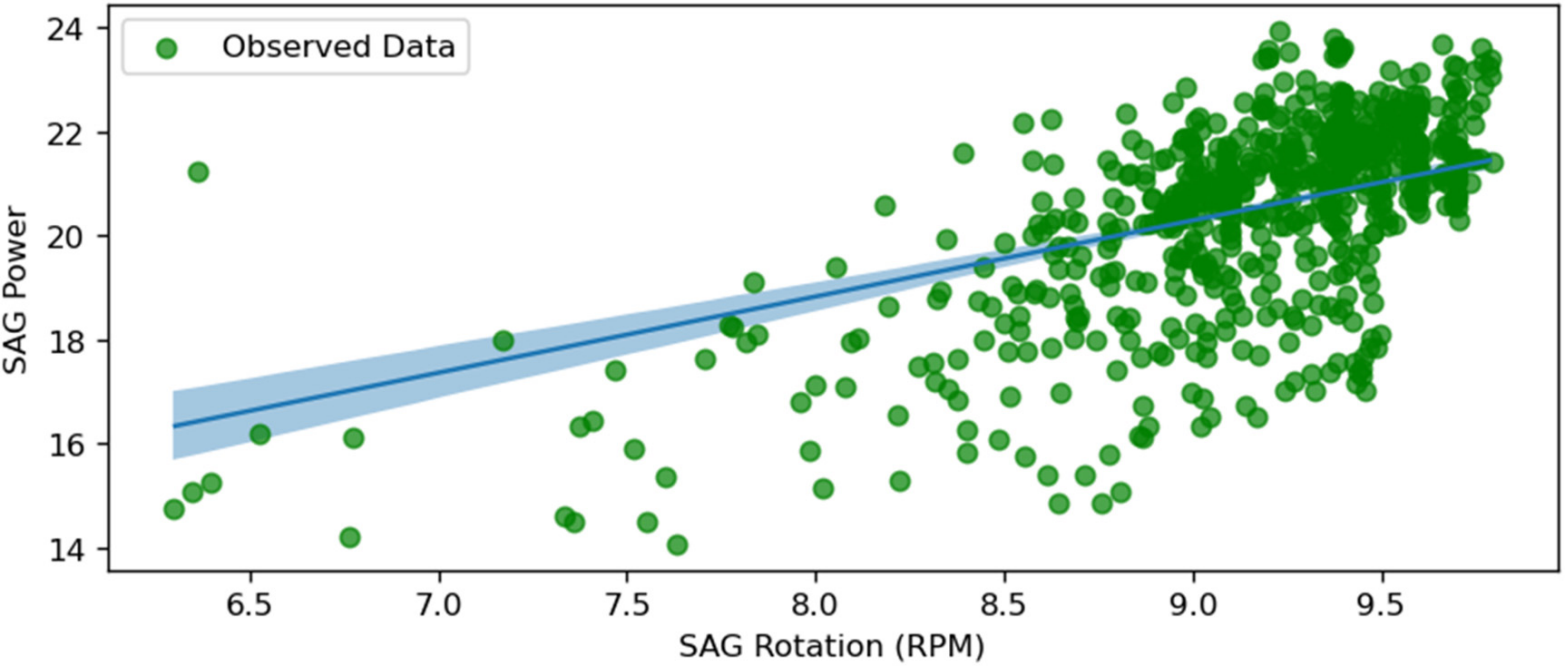
SAG power and SAG RPM.

**Figure 15.**
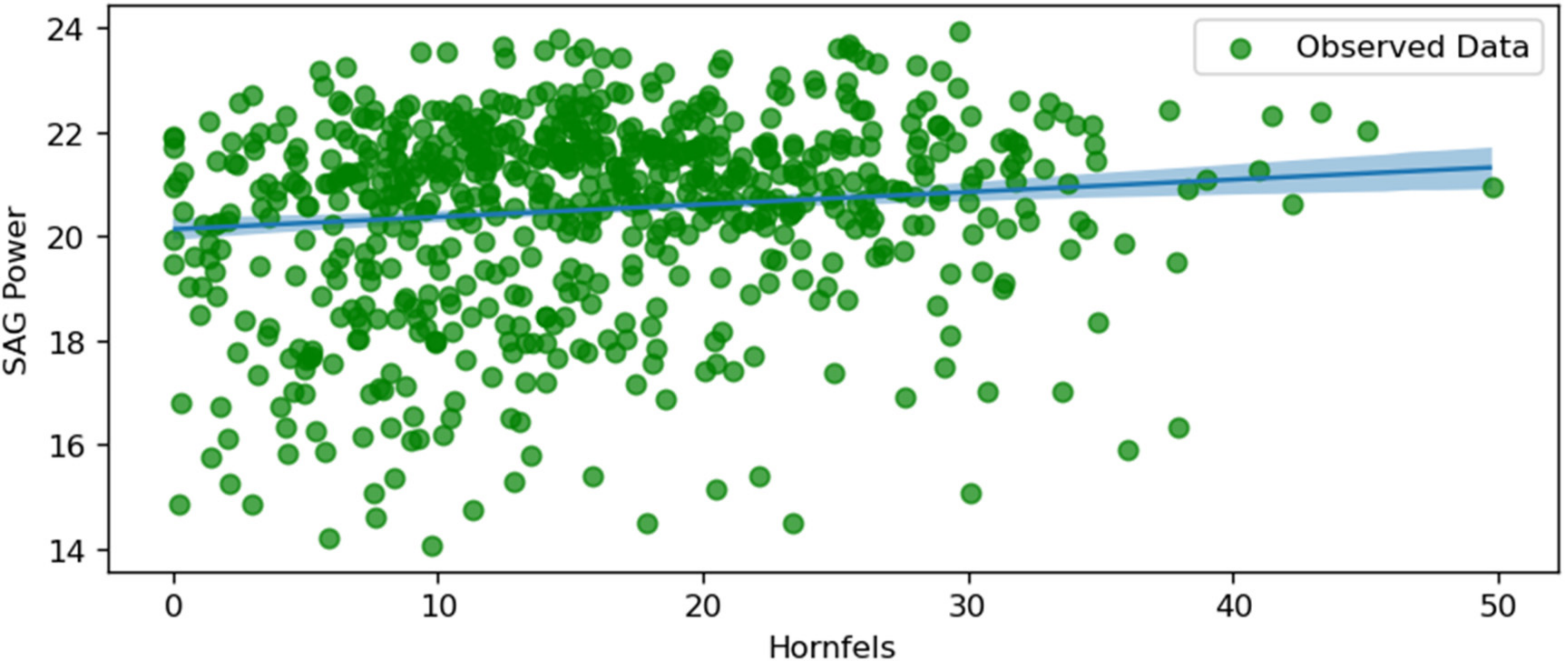
SAG power and hornfels.

**Figure 16.**
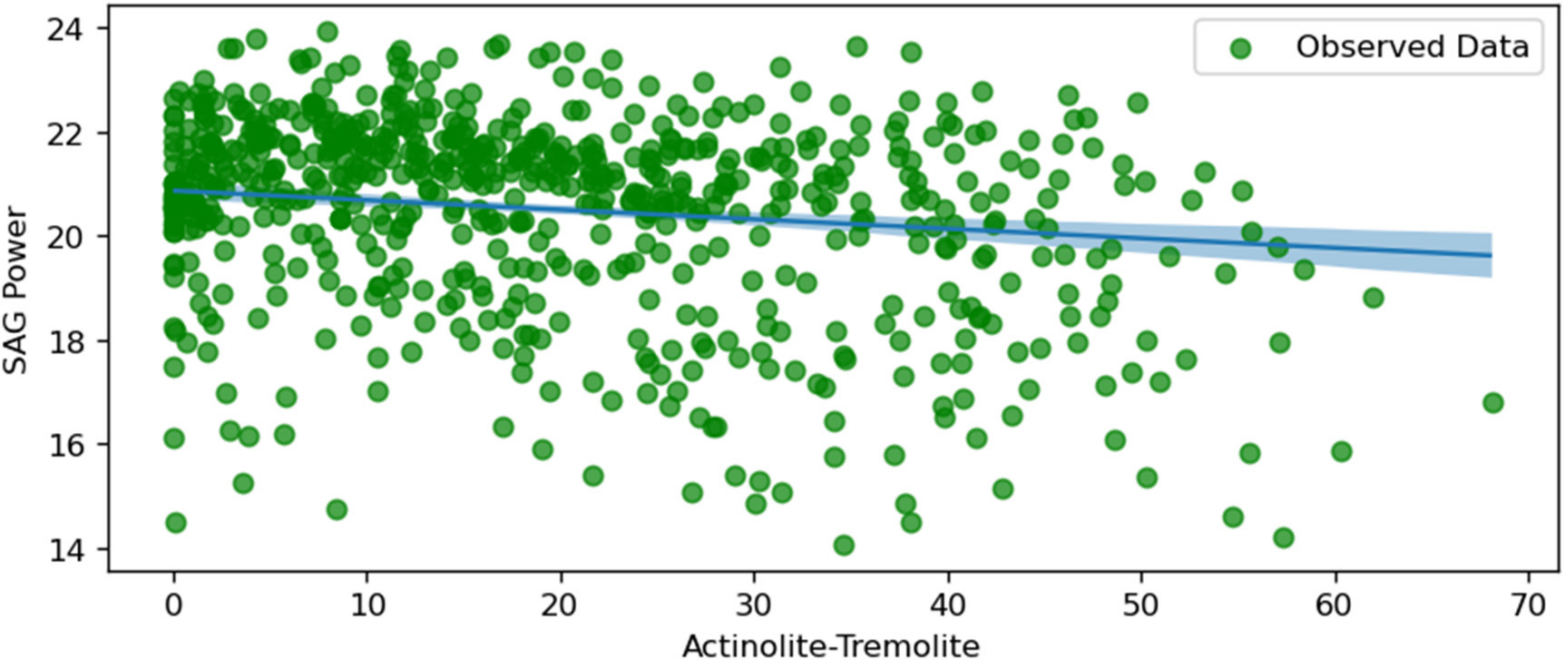
SAG power and actinolite tremolite.

**Figure 17.**
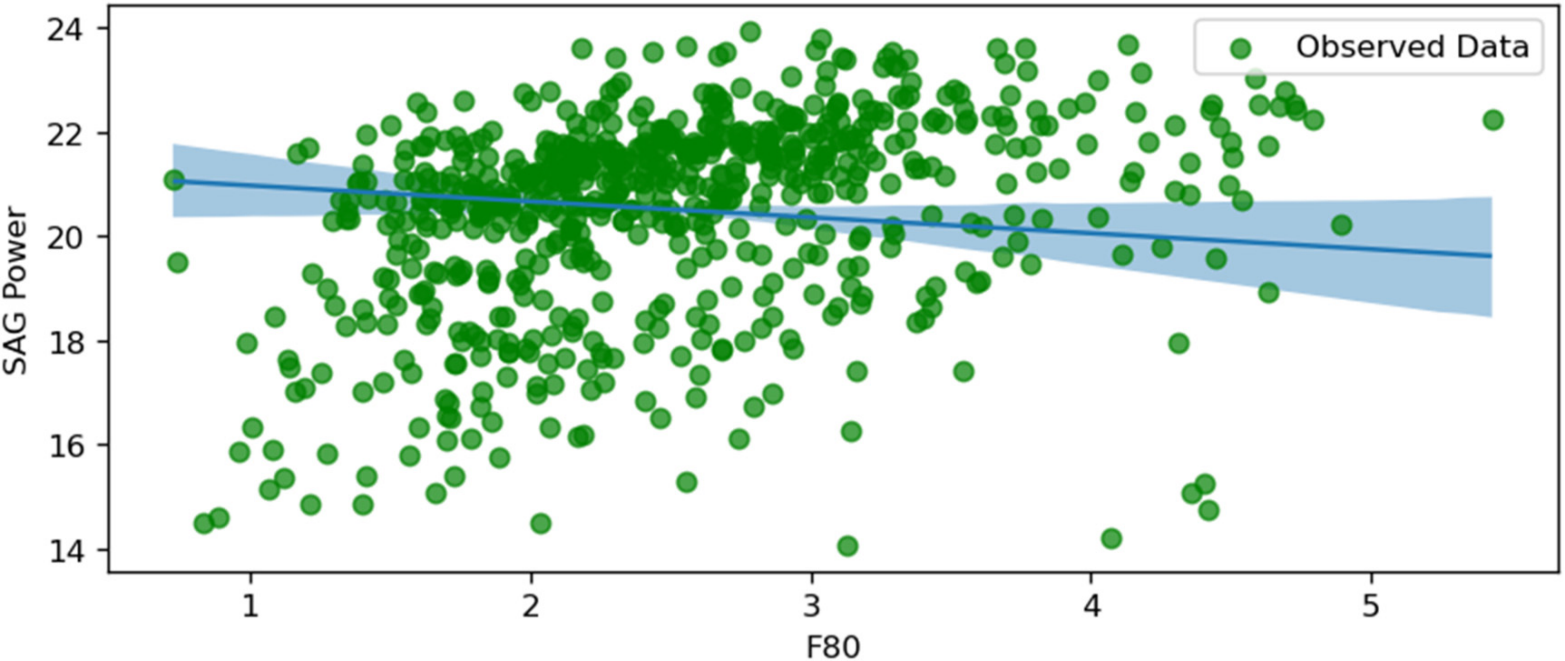
SAG power and F80.

The following figures, starting from Figures 10 to 17, visualise regression models by plotting conditionally adjusted predictions. They show how a chosen parameter of the response distribution varies with interpolated explanatory variables. The posterior mean and HDI are displayed. In Bayesian statistics, a 94% HDI represents the highest probable density, encompassing 94% of the distribution. The panels show the posterior mean and 94% Highest Density Interval (HDI). HDI provides the highest probable density, encompassing 94% of the distribution. Ninety-four per cent is an arbitrary choice; it is used in Bayesian statistics compared to 95% in frequentist statistics. It offers a balance of robustness and coverage.

The 94% HDI shaded area shows the uncertainty around the predictions. This kind of visualisation is helpful for implementing and facilitating mine-to-mill strategies to control variables from different domains. The following plots are useful for analysing the regression models by plotting conditional predictions. This feature visualises how the adjusted response distribution varies because of the interpolated explanatory variables.

Please note that the figures provided individually may not reveal strong or meaningful relationships. However, when analysed collectively or as part of a group analysis, the variables exhibit significance and contribute to understanding the energy consumption dynamics of the SAG mill. These plots are particularly valuable as they simplify and clarify the role of each variable in the Bayesian regression model by isolating its individual effects on SAG power consumption. While showcasing individual relationships, they also account for uncertainty, as represented by the 94% HDI. This aligns with the broader framework of multivariable relationships, where some variables that seem irrelevant individually may play a crucial role when interactions and dependencies are considered. By providing actionable insights into key drivers, these plots enhance the interpretability and practical implications of the study, supporting informed decision-making for operational optimisation.

Figure 10 illustrates the relationship between SAG power consumption and copper grade (Cu). The shaded area represents the 94% Highest Density Interval (HDI), providing a measure of uncertainty around the predicted values. This suggests that higher copper concentrations in the feed result in more efficient grinding processes, requiring less power.

The plot shows a negative relationship, indicating that as the percentage of copper grade in the ore increases, the power consumption of the SAG mill decreases. The shaded area is less uncertain in the grade range between 0.6 and 0.8%, which means the uncertainty in this range is less than in other parts. According to the normal distribution from Figure 4, the dataset has the most data within this given range.

Figure 11 depicts the relationship between SAG power consumption and the size of intermediate particles (between fines and coarse). The plot shows a positive correlation, indicating that as the size of intermediate particles increases, the power consumption of the SAG mill also increases. This positive relationship suggests that larger intermediate particles require more energy to grind, thereby increasing the mill's power consumption. The difference between uncertainty levels is almost insignificant in the whole plot. Despite the known relationship between these two parameters, the exact percentage or size of intermediate particles could be calculated computationally to minimise the energy consumption values.

Figure 12 shows the relationship between SAG power consumption and throughput (t/h). The plot demonstrates a positive correlation, where increased throughput leads to higher power consumption. This relationship indicates that higher material throughput in the SAG mill necessitates greater power to maintain efficient grinding, reflecting the energy-intensive nature of handling larger volumes of material.

Using a similar Bayesian analysis, the relationship of the optimal throughput value could be established to keep a balance between energy consumption and production throughput. The HDI level is minimal from 4000 t/h to 5000 t/h throughput.

Figure 13 illustrates the relationship between SAG power consumption and the hardness of the ore. The plot shows a positive correlation, suggesting that as the ore hardness increases, the SAG mill's power consumption also rises. This positive correlation implies that harder ore requires more energy to grind, which is consistent with the expected behaviour in mineral processing, where harder materials are more resistant to comminution. This graph is highlighted due to the high uncertainty along the studied variables.

Opposite to the previous figure, Figure 14 shows the least uncertainty. This figure depicts the relationship between SAG power consumption and the rotation speed of the SAG mill (SAGRPM). The plot shows a strong positive correlation, indicating that higher rotation speeds result in increased power consumption. This relationship suggests that increasing the mill's rotation speed significantly impacts power usage, emphasising the importance of optimising rotational speed to balance energy consumption and grinding efficiency. SAG rotation speed is optimal for practice ranging between 6 and 10 r/min, confirming the model accuracy.

Figure 15 illustrates the relationship between SAG power consumption and the proportion of Hornfels in the ore feed. There is a positive trend, indicating that as the percentage of Hornfels increases, the SAG power consumption also rises. Hornfels, being a harder metamorphic rock, likely requires more energy for comminution, thus driving up power usage. The shaded area represents the uncertainty range, and it grows slightly wider at higher percentages of Hornfels, which may reflect limited data or higher variability in this range. This relationship highlights the importance of considering geological factors like Hornfels content when optimising mill energy consumption.

Figure 16 shows a negative correlation between SAG power consumption and the percentage of Actinolite-Tremolite in the ore feed. As the proportion of Actinolite-Tremolite increases, the SAG power consumption decreases. This suggests that ores containing higher levels of Actinolite-Tremolite may be easier to grind, possibly due to their mineralogical characteristics. The uncertainty range is relatively stable across the plot, with slightly more confidence in the middle range of Actinolite-Tremolite percentages. This insight can help in planning feed compositions to minimise energy consumption during grinding.

Figure 17 depicts a negative relationship between SAG power consumption and F80, which represents the feed particle size through which 80% of the material passes. As F80 increases, SAG power consumption decreases, likely due to a reduced grinding load caused by the predominance of fines (approximately 70% of F80 values). The uncertainty is lower in the middle range of F80 values but increases at the extremes, reflecting reduced data points or higher variability. This relationship underscores the importance of feed size distribution in optimising SAG mill energy efficiency and suggests that coarser feed sizes can result in lower power consumption, provided the mill operates efficiently.

The figures collectively provide insights into the key factors influencing SAG mill power consumption. The negative correlations observed with the copper grade, Actinolite-Tremolite, and F80 suggest that richer ore, higher proportions of Actinolite-Tremolite, and coarser feed sizes require less energy for processing. Conversely, the positive correlations with Hornfels content, intermediate particle size, throughput, ore hardness, and rotation speed underscore the increased energy demands associated with harder materials, larger intermediate particle sizes, and higher operational speeds. These visualisations and analyses are critical for mine operators to optimise SAG mill operations by balancing power consumption with processing efficiency, ultimately achieving cost-effective and energy-efficient outcomes.

Energy consumption in comminution processes is significantly higher than the cost of the energy provided by blasting processes. This is especially true given that the feed size distribution is a variable that could be modified in plant operations and throughput. Powell et al. (2001) stated that throughput can be changed by high mill loading. Therefore, including data representing rock quality, fragmentation, and processing plant variables helps to improve energy consumption predictions by understanding the inner dynamics of the variables.

These analyses aid in controlling parameters and selecting accurate production conditions, such as optimising the rotation of the SAG mill. Achieving the ideal rotation speed and maintaining the appropriate particle size are crucial factors. These models can provide insights into the relationships between variables, ultimately enhancing the production cycle.

### *Random forest*

Unlike Bayesian methods, the RF is helpful to forecast the required output accurately. It shows the variable importance; however, it does not explain due to its different nature. RF constructs multiple decision trees during training and outputs the mean prediction of the individual trees. This approach helps to overcome the limitations of single decision trees, reducing overfitting and improving generalisation, making it particularly suitable for datasets with numerous variables and non-linear relationships.

For this case study, an RF model was employed to predict SAG mill power consumption using a dataset of operational variables. The process of setting up and training the model involved the following steps:

1. Data preprocessing: The dataset was cleaned and preprocessed to handle any missing values or anomalies.
2. Feature selection: Key features such as Intermediate, Hardness, Throughput, Cu, SAGDisPressure, Fines, Pebbles, Hornfels, Act_Trem, Intrusive_A, F80, TonCrushing, Coarse and SAGRPM were identified as the most important variables for model training. These features were selected based on their relevance to the prediction of power consumption in SAG mills. The same pull of variables was chosen to compare the results with the Bayesian method.
3. Model training: The RF model was trained on these selected features using 80% of the dataset, while the remaining 20% was used for testing and validation. The model parameters were optimised using grid search and cross-validation to achieve the best performance. Cross-validation ensures that the model generalises well to unseen data, while grid search helps to fine-tune the hyperparameters.

To achieve the best accuracy for the model, Grid Search with Cross Validation (CV) was applied to systematically explore and evaluate combinations of hyperparameters. The Grid Search optimised the random forest model's performance by identifying the best parameters. This process ensured that the model achieved a balance between predictive power and generalizability. The optimal hyperparameters identified through this approach are presented in Table 2.

**Table 2.** Hyperparameters of the RF model.

Number of estimators	500
Maximum depth	15
Max features	None
Min sample leaf	1
Max sample split	2
Bootstrap	True

These parameters were chosen to balance the model's complexity and performance. A large number of estimators (500) ensures more decision trees are built, capturing more patterns in the data. The maximum depth (15) and other parameters prevent the model from overfitting while allowing sufficient complexity to capture relationships in the dataset.

The performance of the RF model was evaluated on the test set, yielding an *R*² value of .75, a Mean Absolute Error (MAE) of 0.631, and a Root Mean Squared Error (RMSE) of 0.900. The *R*² value indicates that 75% of the variance in the target variable (SAG mill power consumption) is explained by the model. These results demonstrate a significant improvement in the model's ability to explain variability compared to earlier iterations.

Feature importance in an RF model indicates how much a given variable contributes to the predictions. The model's feature importance is listed in Table 3.

**Table 3.** Feature importance results.

Variable	Importance
SAG RPM	0.51
SAGDisPressure	0.21
Fines	0.09
Pebbles	0.03
Throughput	0.02
Hornfels	0.02
Cu	0.02
Act_Trem	0.02
Intrusive_A	0.02
Hardness	0.01
Intermediate	0.01
F80	0.01
Ton crushing	0.01
Coarse	0.01

**Figure 18.**
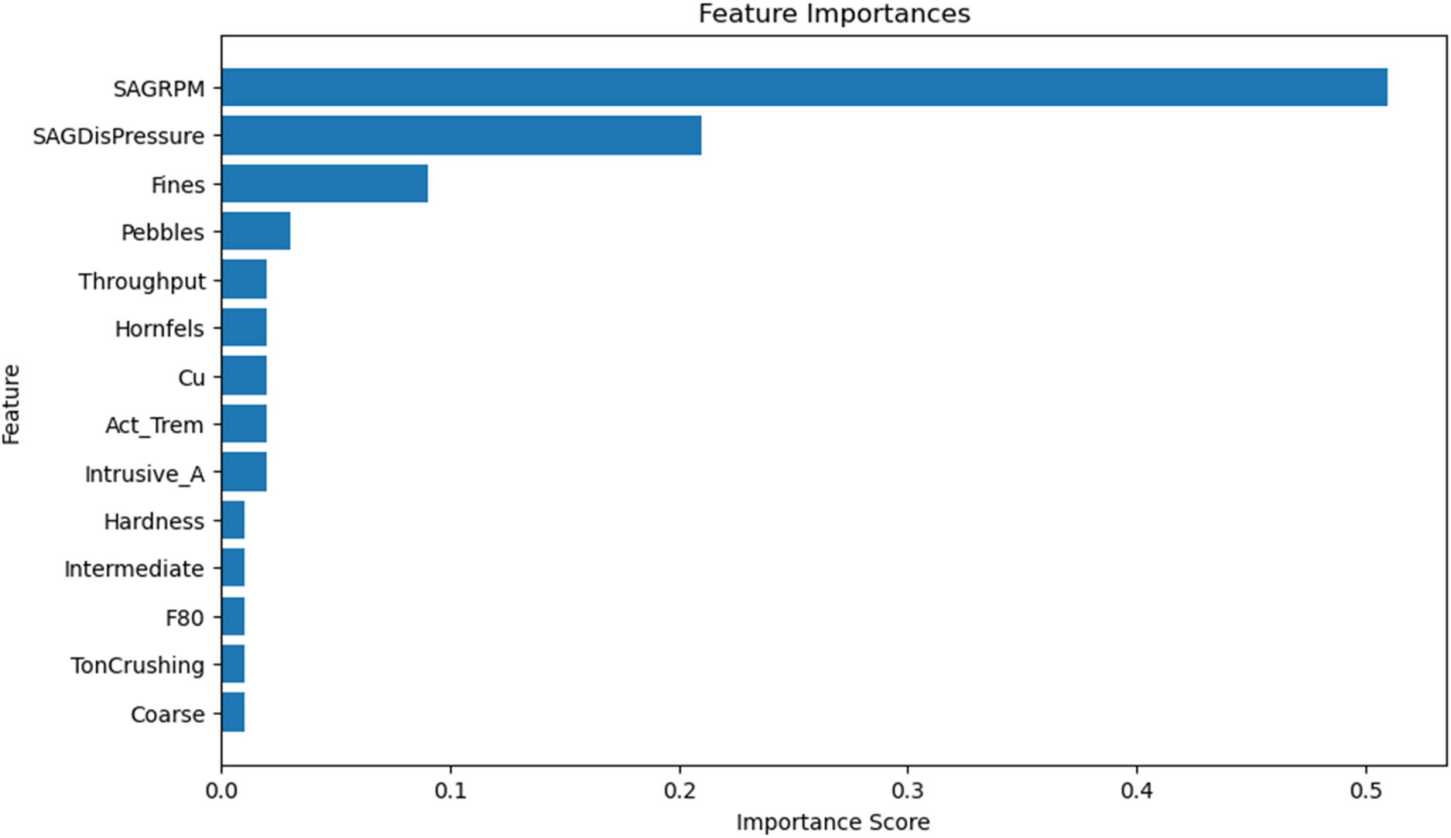
Feature importance.

As shown in the feature importance in Figure 18, SAGRPM is by far the most important variable, contributing 51% to the model's predictions. This aligns with prior domain knowledge and Bayesian GLM results, where SAGRPM was also identified as the most significant predictor. SAGDisPressure and Fines play a secondary but notable role, while Pebbles, Throughput, Hornfels, Cu, Act_Trem, and Instrusive_A have smaller but still relevant contributions to the prediction.

**Figure 19.**
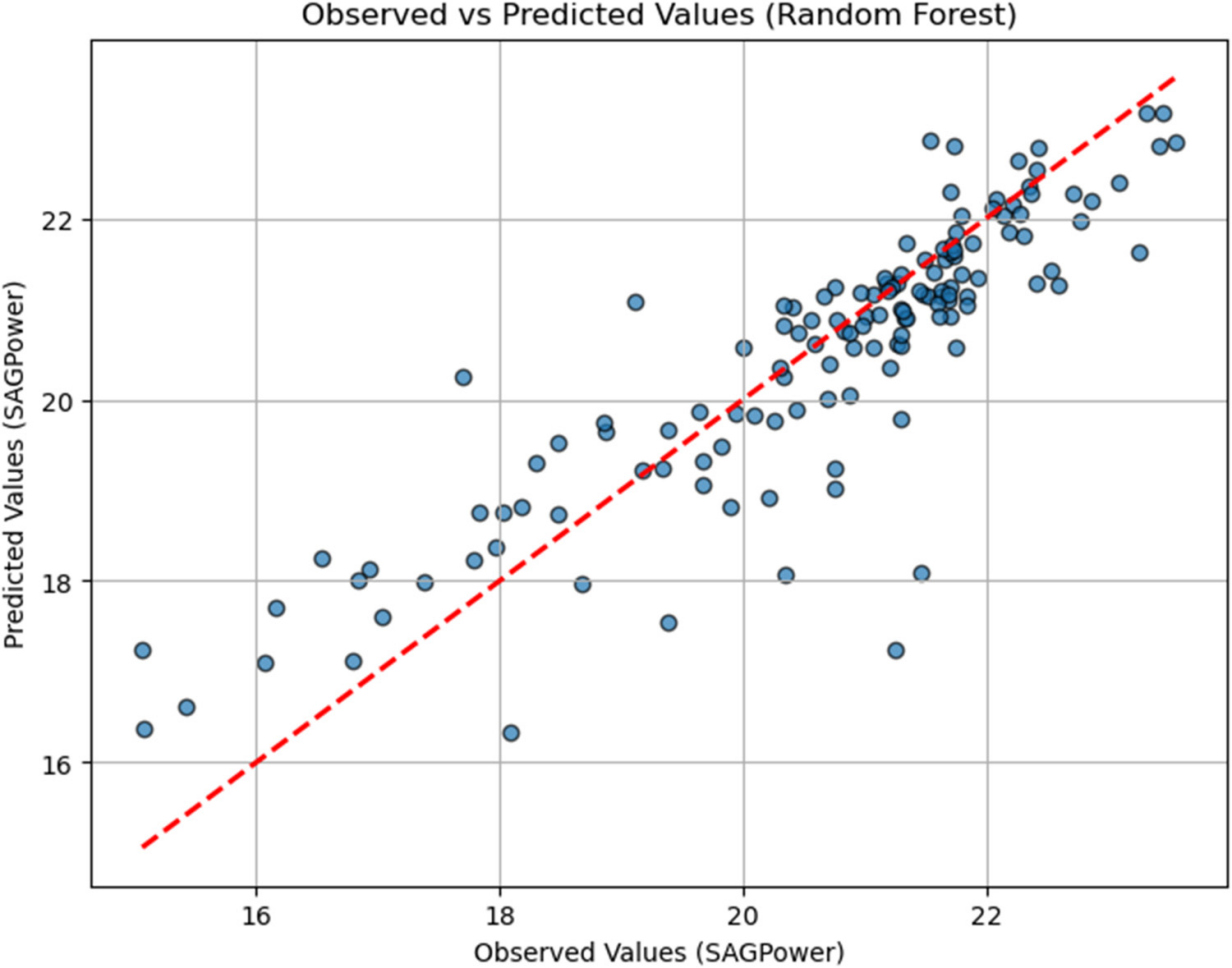
RF performance scatter plot.

To assess the performance of the RF model, a scatter plot comparing observed and predicted values of SAG mill power consumption was created. In the plot shown in Figure 19, the observed values (actual measurements from the dataset) are displayed along the horizontal axis, while the predicted values, generated by the RF model, are plotted on the vertical axis.

The red diagonal line represents perfect predictions, where the predicted values exactly match the observed values. Points closer to this line indicate higher predictive accuracy, demonstrating the model's ability to capture the underlying patterns in the data. Deviations from the line represent prediction errors, providing a visual insight into the model's strengths and limitations.

This plot highlights the RF model's reliability in predicting SAG mill power consumption, reinforcing its suitability for this specific dataset and application.

To facilitate a direct comparison between the RF and Bayesian GLM approaches, Table 4 presents their respective predictive performance and model characteristics. For RF, results are based on the held-out test set (80/20 split), while for the Bayesian GLM, the model was evaluated using LOO to obtain the ELPD. This allows both models to be compared using their most appropriate evaluation metrics while highlighting their complementary strengths.

**Table 4.** Comparison of RF and Bayesian GLM models.

	RF	Bayesian GLM
Metric	*R*^2^ = 0.75	ELPD = −1134.45 (SE = 21.82)
MAE	0.631	Not applicable
RMSE	0.90	Not applicable
Uncertainty quantification	Not applicable	Yes
Data split	80/20 (Train/Test)	Full dataset (via LOO)

## Discussion

Both Bayesian GLM and RF provide valuable insights into the factors influencing SAG mill power consumption, but they approach the problem from different perspectives. The RF model demonstrated strong predictive performance, achieving an *R*² of .75 on the validation set. Evaluation metrics such as an MAE of 0.631 and an RMSE of 0.900 on the validation set highlight the model's reliability and robustness in predicting SAG mill energy consumption. However, while RF effectively captures complex, non-linear patterns, it does not inherently explain the underlying relationships and uncertainties in the dataset.

In contrast, the Bayesian approach provides a detailed understanding of the relationships between variables and their uncertainties. By generating posterior distributions for each parameter, the Bayesian GLM allows for probabilistic interpretations, which are particularly useful when dealing with uncontrollable variables, such as geological properties. This approach supports informed decision-making by simulating both likely outcomes and extreme scenarios, offering a comprehensive view of potential risks and variability in SAG mill power consumption. Furthermore, the ability to incorporate prior knowledge makes Bayesian models highly adaptable to different mining operations.

In this study, both methods identified key variables influencing SAG mill power consumption, including SAG mill rotation speed, intermediate particle size, throughput, copper grade, and ore hardness. The tool Kulprit facilitated efficient variable selection for the Bayesian model, focusing on the most impactful predictors while excluding less significant variables such as Intrusive_A, SAG discharge pressure, and pebbles.

While each method has its strengths and limitations, we emphasise that neither method is inherently superior to the other. Instead, we believe that these two approaches are highly complementary and can improve each other when used together. RF excels at detecting complex, non-linear interactions and making accurate predictions, while Bayesian GLM enhances interpretability by providing insights into parameter relationships and quantifying uncertainties. When combined, these methods offer a robust framework for understanding and optimising SAG mill energy consumption.

By leveraging RF for its predictive accuracy and Bayesian GLM for its interpretability and uncertainty quantification, operators can gain a more comprehensive understanding of the factors influencing energy consumption. This dual approach supports balanced decision-making, ensuring both precision and actionable insights for improving SAG mill operations and energy efficiency.

## Conclusion

This work elucidated the frequentist and Bayesian methods to explore the relationship between operational variables and predict SAG mill energy consumption. The workflows of RF techniques and Bayesian GLM were applied in a case study example, identifying five critical variables – SAG mill rotation speed, throughput, intermediate particle size, copper grade, and ore hardness – as significant predictors of energy consumption.

The analysis highlights that Bayesian GLM and RF provide valuable insights into SAG mill energy consumption. The Bayesian model offers a probabilistic framework that captures uncertainties and provides parameter distributions and credible intervals, allowing for a deeper understanding of the relationships between variables. The RF model demonstrated strong predictive performance, achieving an *R*² of .75 on the validation set and offering robust predictions of energy consumption. However, RF lacks the interpretability and uncertainty quantification that the Bayesian approach provides.

Although SAG rotation speed, ore particle size, and hardness are well-established as crucial predictors of energy consumption, the Bayesian approach adds value by providing probabilistic predictions and plausible scenarios. By applying these complementary techniques, mining operations can gain a holistic understanding of key parameters, assess the impact of interacting variables, and develop strategies that quantify risks and adapt to changing conditions.

The combined use of RF and Bayesian GLM demonstrated that these methodologies are not competing but complementary. Together, they offer a robust framework for predictive modelling and decision-making, combining the predictive accuracy of RF with the interpretability and uncertainty quantification of Bayesian GLM. This synergy enables mining operators to optimise SAG mill operations, balance energy consumption with processing efficiency, and achieve cost-effective outcomes.

The application of ML and Bayesian strategies can be particularly impactful in the following areas:

(1) Mine-to-mill optimisation: Computational models can be integrated to streamline the entire mining process from extraction to processing, improving overall efficiency and reducing costs.
(2) Geometallurgy: ML and Bayesian hierarchical algorithms can analyse geological and metallurgical data for better resource estimation and more efficient processing techniques.
(3) SAG Mill Parameter Control. SAG mill parameters, such as rotation speed and feed size, could enhance energy efficiency and reduce operational costs by applying Bayesian and ML models.

Future work should consider the application of Bayesian hierarchical models, which can provide a more nuanced understanding of the multi-level structure of mineral processing data. The integration of the liner's age with hierarchical models presents a promising avenue for future research, potentially leading to significant advancements in resource estimation and process optimisation.

The transformative potential of Bayesian and ML methodologies in the mining industry has been highlighted in this study. By leveraging these advanced techniques, mining companies can achieve greater operational efficiency, better compliance with environmental regulations, and improved community relations, thereby securing a sustainable future in the challenging landscape of modern mining.
